# Soil mycobiomes in native European aspen forests and hybrid aspen plantations have a similar fungal richness but different compositions, mainly driven by edaphic and floristic factors

**DOI:** 10.3389/fmicb.2024.1372938

**Published:** 2024-05-07

**Authors:** Elisabeth Rähn, Reimo Lutter, Taavi Riit, Tea Tullus, Arvo Tullus, Leho Tedersoo, Rein Drenkhan, Hardi Tullus

**Affiliations:** ^1^Chair of Silviculture and Forest Ecology, Institute of Forestry and Engineering, Estonian University of Life Sciences, Tartu, Estonia; ^2^Department of Botany, Institute of Ecology and Earth Sciences, Faculty of Science and Technology, University of Tartu, Tartu, Estonia; ^3^Mycology and Microbiology Center, University of Tartu, Tartu, Estonia

**Keywords:** *Populus* (hybrid poplar), plantation vs. forest, ectomycorrhizal symbiosis, plant pathogens, regeneration impact, EcM exploration types, first-rotation plantation diversity, former land use impact

## Abstract

**Background:**

The cultivation of short-rotation tree species on non-forest land is increasing due to the growing demand for woody biomass for the future bioeconomy and to mitigate climate change impacts. However, forest plantations are often seen as a trade-off between climate benefits and low biodiversity. The diversity and composition of soil fungal biota in plantations of hybrid aspen, one of the most planted tree species for short-rotation forestry in Northern Europe, are poorly studied.

**Methods:**

The goal of this study was to obtain baseline knowledge about the soil fungal biota and the edaphic, floristic and management factors that drive fungal richness and communities in 18-year-old hybrid aspen plantations on former agricultural soils and compare the fungal biota with those of European aspen stands on native forest land in a 130-year chronosequence. Sites were categorized as hybrid aspen (17–18-year-old plantations) and native aspen stands of three age classes (8–29, 30–55, and 65-131-year-old stands). High-throughput sequencing was applied to soil samples to investigate fungal diversity and assemblages.

**Results:**

Native aspen forests showed a higher ectomycorrhizal (EcM) fungal OTU richness than plantations, regardless of forest age. Short-distance type EcM genera dominated in both plantations and forests. The richness of saprotrophic fungi was similar between native forest and plantation sites and was highest in the middle-aged class (30–55-year-old stands) in the native aspen stands. The fungal communities of native forests and plantations were significantly different. Community composition varied more, and the natural forest sites were more diverse than the relatively homogeneous plantations. Soil pH was the best explanatory variable to describe soil fungal communities in hybrid aspen stands. Soil fungal community composition did not show any clear patterns between the age classes of native aspen stands.

**Conclusion:**

We conclude that edaphic factors are more important in describing fungal communities in both native aspen forest sites and hybrid aspen plantation sites than forest thinning, age, or former land use for plantations. Although first-generation hybrid aspen plantations and native forests are similar in overall fungal diversity, their taxonomic and functional composition is strikingly different. Therefore, hybrid aspen plantations can be used to reduce felling pressure on native forests; however, our knowledge is still insufficient to conclude that plantations could replace native aspen forests from the soil biodiversity perspective.

## Introduction

Short-rotation forestry (SRF) is a silvicultural system to supply wood for the bioeconomy (Szuba, 2015); when established on former agricultural land, it helps to reduce harvest rates in native forests (Weih, 2004; Tullus et al., 2013). *Populus* species are important for energy production in many regions due to their low need for nitrogen, compared with other potential energy crops, such as corn or agave (Somerville et al., 2010). Among the suitable tree species, hybrid aspen (*Populus tremula* L. × *P. tremuloides* Michx., syn. *P.* × *wettsteinii*) is one of the most widely planted tree species for plantation forestry in Northern Europe (Tullus et al., 2012; Hytönen et al., 2020; Fahlvik et al., 2021; Lee et al., 2021), where its production is more than double that of native European aspen stands of the same age (Rytter and Stener, 2014; Lutter et al., 2021). Additionally, planting hybrid aspen and fast-growing willow cultivars is considered as a way for sustainable bioenergy production (Davis et al., 2012) and climate change mitigation (Rytter and Rytter, 2020a; Lutter et al., 2021).

The general approach of hybrid aspen management is similar to intensive forest plantation management as hybrid aspen stands are regenerated by clearcutting and managed with relatively short rotation cycles (25 years) in comparison with conventional forestry (Tullus et al., 2012). Therefore, it has been argued that such plantations are “green deserts” that host low biodiversity (Horák et al., 2019; DellaSala, 2020). Contradicting this view, a number of studies have found that hybrid aspen plantations on previously agricultural land have great potential to host vascular plant, bryophyte, and lichen species early in their development due to their rapid growth but sparse canopy (Tullus et al., 2015; Randlane et al., 2017), particularly if plantations are located in the vicinity of forests as possible sources of colonization (Randlane et al., 2017). However, the diversity and communities of soil fungal organisms, which are important drivers of forest health, biomass productivity (Van der Heijden et al., 2008), and nutrient cycling (Treseder and Lennon, 2015; Bahram et al., 2020), are poorly known for former agricultural lands afforested with hybrid aspen short-rotation coppice. Nevertheless, understanding biodiversity and shifts in soil properties and biota in plantations is necessary, as plantations could eventually host and therefore preserve numerous taxa by enlarging their habitat, and thus enable their spread to further areas. Hence, afforestation may help to reduce forest fragmentation caused by forest losses in smaller regions (e.g., Baltic states). Moreover, tree growth and health depend on microbial communities. Usually, older forest stands are considered more valuable or even irreplaceable regarding biodiversity when compared to recultivated stands, young forests, or plantations. Older stands also host late-successional fungi that are scarce or absent in newly established sites where ectomycorrhizal (EcM) fungi with an early-successional strategy (or pioneers) are better adapted to colonize new areas (Vlk et al., 2020). Clearly, the effect of stand age on estimates of average species richness depends on the studied species groups (Tullus et al., 2022b; Rähn et al., 2023). Also, the importance of stand age in describing biodiversity is linked to forest structural attributes (Crites and Dale, 1998), which are just as important when preserving biodiversity.

European aspen (*Populus tremula* L.) is economically one of the most important deciduous tree species in Northern Europe. It is also considered one of the most important key species for biodiversity in boreal forests (Hardenbol et al., 2020) as it hosts a wide variety of epiphytic bryophytes and lichens, polypore fungi, invertebrates, birds, and animals, including many red-listed species (Kivinen et al., 2020). Other aspen species (e.g., *Populus tremuloides*, *P. davidiana*) likewise form biodiversity hotspots in the landscape of conifer dominated forests of the Northern Hemisphere (Rogers et al., 2020). However, the influence of monospecific poplar (*Populus* spp.) plantations on biodiversity is often considered to be negative due to the low genetic and habitat variability of plantations (Rotach, 2004).

In Sweden, European aspen regeneration has been declining since the 1970s due to land use change and mechanical tree removal (Edenius et al., 2010), with adverse impacts on aspen-related species (Kouki et al., 2004). At the same time, the area covered by hybrid aspen plantations has increased in the region (Xu and Mola-Yudego, 2021), while knowledge about soil microbial communities of hybrid aspen plantations continues to be scarce in comparison with that of native aspen stands. As hybrid aspen plantations are mainly established on previous agricultural land, differences in fungal communities between plantations and native aspen forests can be expected, in line with the results of Balami et al. (2020). On the other hand, some studies have claimed that plantation soil fungal communities could be similar to native forest ones, but the time range considered varies considerably between studies. A study in Canada (Yannikos et al., 2014) found that microbial communities developed toward native aspen forest communities in 7- and 14-year-old hybrid poplar (*Populus deltoides* × *P petroskyana*) plantations on former agricultural land. Jangid et al. (2011) reported that after 50 years, deciduous afforested cropland microbial communities became more similar to those of the forest stands. According to another study, after three decades of Chinese pine plantation development the soil microbial structure had become relatively stable and similar to that of native undisturbed pine stands (Dang et al., 2017). A comparison of silver birch plantations and naturally regenerated stands on former agricultural soils with birch stands on native forest land did not show any differences in fungal richness but revealed compositional differences (Lutter et al., 2023) as fungal community compositions in birch stands on forest land were significantly different from stands on former agricultural land.

The afforestation of agricultural land is a considerable land use change, where trees can alter soil physical and chemical properties (Augusto et al., 2002; Berthrong et al., 2009). The legacy of former agricultural land use on soil chemical properties after afforestation can persist for several decades compared to native forests (Wall and Hytönen, 2005; Falkengren-Grerup et al., 2006; Tullus et al., 2022a). According to current knowledge about the effects of deciduous forest plantations on former agricultural soil, there are no major changes in soil carbon and nutrient pools 15–20 years after afforestation (Lutter et al., 2016a,b, 2023). However, there is a significant decrease in soil pH (Lutter et al., 2016b; Rytter and Rytter, 2020b), a key driver of fungal communities (Tedersoo et al., 2020). Even after long periods plantation soil remains different from forest soil, and such dynamic changes in soil properties can also affect soil fungal communities (Koorem et al., 2014).

Afforestation creates a new biotope that could host species from forest soils such as ectomycorrhizal fungi (Baum et al., 2009). EcM fungi are vital for tree growth. Aspen and poplar species interact with both EcM and arbuscular mycorrhizal (AM) fungi. AM fungi are more diverse and widespread in grass- and croplands but suppressed in hemiboreal forests due to EcM plant abundance, as discussed in Ferlian et al. (2021).Yet the ratio of EcM and AM fungi varies by *Populus* species and hybrid (Karliński et al., 2010). Nevertheless, the dominative fungal mutualists with (European) aspen are EcM fungi (Lodge and Wentworth, 1990; Kaldorf et al., 2004; Krpata et al., 2008) thus it can be expected that EcM fungi dominate over AM fungi shortly after afforestation of grass- or croplands with hybrid aspen. EcM fungi also have a beneficial effect on poplar growth during afforestation and are important for poplar reproductive potential (Szuba, 2015). EcM fungi may colonize the roots of aspen clones shortly after cultivation, increasing the species richness in young plantations within 1 year (Danielsen et al., 2012). It has been recorded for several tree species, that there is tree-genotype-specific variation in the formation of ectomycorrhizal symbioses and that this variation should be considered in the selection of tree genotypes for breeding (Rosado et al., 1994). Such variation has also been observed among progeny from a cross between *Populus deltoides* and *Populus trichocarpa* (Tagu et al., 2001). Hence, it is necessary to determine which EcM genera dominate in hybrid aspen plantations and native aspen forests and what genera of all major fungal groups are significantly different between these site types. Diverse soil fungal biota can enhance nutrient cycling and stabilize soil carbon (Bahram et al., 2020; Tomao et al., 2020), along with protecting plants from pathogens (Howell, 2003) and environmental stresses (Classen et al., 2015), resulting in more productive plants with better overall growth. EcM fungi control plant acquisition of nutrients, such as nitrogen, carbon, and phosphorous, as they play a crucial role in recycling nutrients (Heijden and Horten, 2009) just as crucially as saprotrophic fungi contribute to decomposition processes. Ability to form *Populus*-ectomycorrhizal symbioses in arable lands (such as hybrid aspen plantations) is a special issue for aspen due to the low content of local EcM fungi and high abundance of pathogenic fungi (Baum et al., 2002). Root colonization by ectomycorrhizal fungi can influence metabolic processes in leaves of Populus species, rendering them more resistant to biotrophic pathogens such as rust-causing Melampsora fungi (Pfabel et al., 2012).

In the current study, we analyze the soil fungal communities in 17–18-year-old hybrid aspen plantations on former agricultural land and in a 131-year chronosequence (young to pole-stage 8–29 years, maturing to mature stands 30–55 years, and 65–131-year-old old stands) of European aspen stands on forest land. Our main objective in this study is to determine which major fungal functional guilds (AM, EcM, plant pathogens, and saprotrophic fungi) and particular genera respond to the site (plantation/forest), soil properties, and stand floristic factors, as well as how previous land-use related and forest management factors affect hybrid aspen soil fungal richness and community composition. Moreover, we aim to describe which fungal genera, particularly EcM taxa and exploration types have successfully colonized first rotation 17–18-year-old hybrid aspen plantation soils.

Spatial distribution analysis (Ramsfield et al., 2020) has shown that undisturbed forests contribute EcM fungi to reclaimed areas via dispersal. Moreover, the study showed that fungal richness could be even greater than that in adjacent undisturbed forests within 10–20 m from the forest edge. EcM fungi in forest ecosystems drive the availability of soil nutrient such as nitrogen (N) and phosphorous (P) to plants (Read and Perez-Moreno, 2003), and this is also the case in plantation forest ecosystems. However, not all EcM fungi respond to soil nutrients the same way (Pellitier and Zak, 2021). EcM exploration types (mainly determined by extraradical mycelium) determine how different taxa colonize new roots, form mycorrhizal networks and forage (Agerer, 2006) which in turn affect nutrient transportation to plants. EcM fungal species of high biomass such as long-distance type are more enriched with nitrogen isotopes (Hobbie and Agerer, 2010) and transport water and nutrients more efficiently than hydrophilic short-, contact- and medium-distance exploration species of EcM fungi (Agerer, 2001). Short-distance or contact types of EcM fungi have a broader environmental range and higher abundance compared to other exploration types (long, medium-smooth and medium-fringe) (Rosinger et al., 2018). EcM fungal successional stages are tied with overall stand development. For example, Twieg et al. (2007) found that host-specific fungi dominated in 5-year-old mixed temperate forests while host generalists were more dominant in older forests.

Considering the latter, we propose that contact and short-distance type genera are the primary EcM foraging type in plantations as well as in forests. We suggest that long-distance species with greater ecophysiological efficiency (Agerer, 2001) exhibit lower abundance in plantations when compared to forests. We also hypothesize that late-successional EcM fungi are absent or scarce in hybrid aspen plantations compared to native aspen forests. We hypothesize that native aspen stands host a higher richness of EcM fungi and different types of EcM genera than hybrid aspen plantations on former grass- and agricultural lands because EcM fungi are not “native” to grasslands. We also hypothesize that the overall fungal richness in soil of hybrid aspen plantations increases with proximity to forests. We also propose that saprotroph and EcM diversity is highest in the oldest age class (65–131-year-old stands) of native aspen stands when compared to younger classes (8–29 and 30–55-year-old stands), since it has been reported that the age of the tree and fungal diversity have a strong positive relation (Tedersoo et al., 2014, 2020).

## Materials and methods

### Study areas

The study sites were scattered throughout southern Estonia (57°33′19″-58°49′46″N, 25°20′51″-27°20′92″E). All sites were located on typical fertile soil, Retisols and Umbrisols (IUSS Working Group WRB, 2015), corresponding to *Oxalis* and *Aegopodium* forest site types (Lõhmus, 1974). According to the closest meteorological station, mean annual precipitation (726 mm) and mean temperature (6.3°C) in the sampling year (2017) were similar to the last 10-year averages of 715 mm and 6.5°C, respectively (Estonian Weather Service, www.ilmateenistus.ee).

The study was performed in 20 hybrid aspen (*Populus tremula* × *P. tremuloides* Michx.) plantations on former agricultural land (grass- or cropland) and 19 native European aspen (*Populus tremula* L.) stands on previously forested land (Supplementary Table S1). The studied hybrid aspen plantations were 17–18 years old, i.e., reaching the final third of their predicted 25-year rotation cycle (Tullus et al., 2012). Hybrid aspen plantations were established with micro-propagated clonal material in 1999 and 2000, using on average 15 different clones per plantation (Tullus et al., 2007). Before the establishment of hybrid aspen plantations, previous land use was as cropland (*N* = 9) or grassland (*N* = 11) (Lutter et al., 2016b). Hybrid aspen sites were located 35–670 m from the nearest forest edge (Tullus et al., 2015). On 13 sites, agricultural land use had ceased more than 1 year before afforestation, and on seven sites, afforestation was performed immediately after field abandonment. The native aspen stands belong to the Järvselja Training and Experimental Forestry District.^1^ The studied native aspen stands presented a chronosequence from the age of 8–131 years that were later divided into three age classes—A_nat: young stands between 8 and 29 years (*n* = 7 plots), B: mature stands between 30 and 55 years (*n* = 6 plots), and C: old stands between 65 and 131 years (*n* = 6 plots). According to the forest inventory, all the native aspen stands grow on forest soils and originate from stump and root sprouts after clear-cutting. Thinning was done in seven hybrid aspen (period 2015–2017) and in five native aspen stands (previous management activities of native aspen stands were determined based on stumps). The data collected in the present study have been previously used or considered in large-scale studies (Tedersoo et al., 2020; Mikryukov et al., 2023); however, the aims and objectives were different and incomparable.

### Field sampling

Soil sampling was carried out according to Tedersoo et al. (2014) on a 2,500-m^2^ circular plot per sampling site. At each site, 40 topsoil subsamples (5 cm diameter to 5 cm depth) were collected from 20 randomly selected trees at a distance of 1–2 m from two opposite sides. A special plastic pipe was used to collect a soil sample after the woody debris and litter were removed from the ground. All 40 subsamples of each site were pooled and set to air dry on a heated floor at 20°C immediately after sampling to minimize mold overgrowth.

Field sampling was carried out during the middle of the growing season (June and July) in 2017 in all studied sample plots. Metadata regarding undergrowth vegetation (richness of vascular plants -including woody and herbaceous species), number of woody understorey species, wood stem volume, basal area, and edaphic characteristics (0–10-cm upper layer) were obtained from previous studies carried out 1–2 years before the soil sampling of the present study (Lutter et al., 2016a,b, 2017; Randlane et al., 2017; Tullus et al., 2022b).

### Soil properties

The chemical properties of each composite sample were measured using 20 g of dried, homogenized soil. Soil acidity (pH_KCl_) was determined in a 1-M KCl solution. Soil-available phosphorous (P, mg kg^−1^) and potassium (K, mg kg^−1^) were extracted with ammonium lactate solution, and available magnesium (Mg, mg kg^−1^) and calcium (Ca, mg kg^−1^) were extracted with ammonium acetate solution (5 g in 100 mL) with shaking for 90 min. The ^13^C and ^15^N stable isotopes (%) were determined with an isotope mass spectrometer (MAT 253; Thermo Electron, Bremen, Germany), and soil total nitrogen (N, %) was measured using the Kjeldahl method (Kjeldahl, 1883). The soil organic matter content was determined via loss on ignition at 360°C for 2 h. The soil moisture content in plantations was categorized according to drainage (auto-, semi-, and hydromorphic soils) (Tullus et al., 2012).

### Molecular analyses and bioinformatics

The dried samples were mixed, crushed by hand, and subsequently homogenized using bead beating with two 3.2-mm diameter stainless-steel beads (BioSpec Products, Bartlesville, OK, United States) in 2-mL tubes containing the sample and the two beads, which were shaken for 5 min at 30 Hz using the Retsch Mixer Mill MM400 (Retsch, Haan, Germany). The DNA was extracted from 2.0 g of soil dust using the Power Max Soil DNA Isolation kit (Qiagen, Carlsbad, CA, United States) following the manufacturer’s instructions. The DNA extracts were further purified using the FavorPrep™ Genomic DNA Clean-Up kit (Favorgen, Vienna, Austria) and subsequently subjected to amplification with the primers ITS9mun and ITS4ngsUni (Tedersoo and Lindahl, 2016). For amplification, the PCR mixture contained 5 μL of 5x HOT FIREPol Blend Master Mix (Solis Biodyne, Tartu, Estonia), 0.5 μL of each forward and reverse primer (20 mM), 1 μL of DNA extract, and 18 μL of ddH_2_O. Thermal cycling included an initial denaturation at 95°C for 15 min, 25–30 cycles of denaturation for 30 s at 95°C, annealing for 30 s at 57°C, elongation for 1 min at 72°C, and final elongation at 72°C for 10 min; the samples were stored at 4°C. The PCR products were normalized for library preparation and sequenced on a PacBio Sequel instrument using SMRT cell 1 M, v. 2 LR, Sequel Polymerase v. 2.1 and Sequencing Chemistry v. 2.1. Loading was performed by diffusion; one SMRT cell was used for sequencing with a move time of 600 min and a pre-extension time of 45 min. Subsequent quality filtering of FASTQ-formatted reads was performed using PipeCraft 1.0 (Anslan et al., 2017) as described in Tedersoo and Anslan (2019). As a reference database for chimera filtering and identification, the UNITE database was used (Kõljalg et al., 2013). Using VSEARCH, sequences were clustered to operational taxonomic units (OTUs) at 97% sequence similarity to enable the separation of closely related species. The OTU matrices were sorted by negative and positive controls to detect laboratory and technical contamination before statistical analyses. FunGuild (Nguyen et al., 2016) and a beta version (unpublished) of FungalTraits (Põlme et al., 2020) reference databases were used to determine functional groups of fungi. The genera that were missing in these databases (uncovered taxa) were functionally annotated based on literature searches using Google Scholar, and this information was further used to update FungalTraits (Põlme et al., 2020). OTUs were assigned to functional groups according to the dominative/first lifestyle. In case of multiple lifestyles (first and secondary) we researched genera or species of interest further, e.g., *Hypoxylon,* and assigned it to species level. Putative yeasts were always considered saprotrophs. Aspen was considered ectomycorrhizal host since many studies have confirmed that EcM dominates over AM fungi as mentioned by Brundrett and Tedersoo (2018). EcM exploration types were assigned according to Tedersoo and Smith (2013) and the Determination Ectomycorrhizae (DEEMY) database (http://www.deemy.de/; Supplementary Table S2). Genera with multiple foraging strategies were assigned following Agerer (2001) or the DEEMY database, also taking into account previous literature. EcM successional stages of a few genera were identified by Dickie et al. (2013) and Vlk et al. (2020) (Supplementary Table S2). However, assigning EcM fungal succession is best described in the context of overall site development (Twieg et al., 2007) and by resource use and competition (Taylor and Bruns, 1999) and requires separate in-depth research which is not the aim of the present study and is not thoroughly discussed.

### Statistical analysis

#### Fungal richness

For statistical analyses, we used the vegan package (Oksanen et al., 2017) as implemented in R version 4.1.2 (R Core Team, 2021), PRIMER-e^2^ version 7 and STATISTICA 12 (TIBCO Software Inc.), with statistical significance at 95% probability (alpha = 0.05). The fungal richness of all sampling sites (*N* = 39) was computed for major fungal functional guilds (all fungi, saprotrophs, EcM, plant pathogens, and arbuscular mycorrhizae) and separately for hybrid aspen (*N* = 20) and native aspen sites (*N* = 19). For this, we performed a linear regression, where the response variable was the number of OTUs and the explanatory variable a square root-transformed number of sequences (to control variation in sequencing depth) (Tedersoo et al., 2014; Tedersoo and Lindahl, 2016), and used standardized residuals as richness proxies. The relative abundances of fungal guilds were computed by summing the proportions of individual OTU sequences relative to the total number of sequences per sample. In addition to standardized residuals, we also calculated the estimated marginal mean OTU richness values employing the R function *emmeans*, using the total number of reads per sample as the control factor to adjust for variable sequencing depth, to more intuitively display the differences in richness among groups.

The effects of stand type (plantation/native forest land), edaphic, floristic, and other environmental factors (see Supplementary Table S1 for detailed data) on richness were analyzed using general linear modeling (type III). Continuous factors were log-transformed before analyses. Potentially significant covariables were tested with multiple regression before modeling, where each factor with *r* > 0.10 was involved in preliminary models. With the autocorrelation check, we excluded one of the two variables with *r* > 0.5 from the analyses. This decision was informed by previous tests with other groups and/or their effects on other factors. Therefore, when determining which factor to include, we took into account the patterns observed in prior analyses. Categorical factors (thinning, former land use and soil moisture regime), as well as the period during which the land was in use before afforestation (> 1 year or immediate), were tested with one-way ANOVA before modeling to test if the factor was potentially significant. The best predictors were selected with the backward elimination method. We tested the management effect (thinning) with the Kruskal–Wallis test due to uneven sample sizes of plantations (*N*_thinned_ = 7, *N*_unthinned_ = 13) and forests (*N*_thinned_ = 5, *N*_unthinned_ = 14). The effects of former land use and soil moisture regime were studied in plantations. All forest study sites were previously aspen-dominated, i.e., native European aspen stands. The EcM woody understorey species richness (%) was manually determined based on literature data (Supplementary Table S3) and analyzed using multiple regression analysis. If the mycorrhizal type of a woody plant species was missing or unsure according to literature sources, we considered these species as non-ectomycorrhizal or unknown and they were only counted in the percentage of EcM woody understorey richness (%). The effect on the richness and relative abundance of EcM host density (%) based on basal areas (Supplementary Table S1) was analyzed using multiple regression analysis. To test the stand age group effect, we formed the following four groups with similar sample sizes: we randomly selected seven hybrid aspen stands aged 17–18 years (A_hyb) and divided the native aspen stands into the following age classes: A_nat: young stands between 8 and 29 years (*n* = 7 plots), B: mature stands between 30 and 55 years (*n* = 6 plots), C: old stands between 65 and 131 years (*n* = 6 plots). The effects of the four age groups on soil fungal richness were tested with one-way ANOVA, followed by Tukey’s *post-hoc* test. The statistics are based on the relative abundances and residual richness values. The *post-hoc* results are reported with estimated marginal OTU richness values to better understand the magnitude of the differences between forest and plantation.

Additionally, we analyzed residual richness and relative abundance of fungi at a genus level to determine significantly different fungal genera between site (plantation/forest) and age groups [A_hyb (hybrid aspen ages 17–18), A_nat (native forest 8–29 y.o), B (native forest 30–55 y.o), and C (native forest 65–131 y.o)]. Selected factors were analyzed because site and age groups were the main questions in this study. Modeling for genus-level proxies was not done, as it was not the goal of this study. Furthermore, since not every EcM exploration (foraging strategy) type associates alike with different environmental aspects, we calculated a proportion of OTUs and sequences of exploration types by site for comparison of plantation and forest. Reported genera-based results in this study are mainly descriptive and are planned to be used later in more specific work that focuses on EcM fungi and their successional stages and exploration types.

#### Fungal community composition

Fungal community composition was analyzed in PRIMER-e (see text footnote 2). The OTU abundance was standardized and square root-transformed by samples before the statistical analyses to reduce the effect of dominant taxa and maximize the model fit. A Bray–Curtis dissimilarity (Bray and Curtis, 1957) matrix was employed as a distance measure. For all fungal group datasets, OTUs with < two sequences (over all samples) and samples < two OTUs were excluded. We performed PERMANOVA+ (Anderson, 2005) (see text footnote 2) for pair-wise analyses on age classes and to detect the significant predictors of fungal community compositions of all sites and plantation and forest sites separately. The best models were selected by the backward elimination method, where potential predictors that described <1% of the variation (based on the adjusted *R*^2^) were eliminated despite the *p*-value. To describe the distribution and possible patterns among communities within each major fungal group, we created principal coordinate analysis (PCO) plots along with vectors of significant predictors (based on the PERMANOVA results) for (1) the overall dataset- (all studied forest and plantation sites) (*n* = 39) categorized by forest and plantation and (2) seven randomly selected samples of plantations for balanced sample size (the same as used in age group-richness section) and three different forest age classes as assigned previously (*n* = 28 and *n* = 25 for AM communities, i.e., the samples that had AM fungi in community composition) for age group analyses. Ordinations showing associations between fungal genera/exploration types and sample groups (site and age classes) were constructed using R functions metaMDS and vectors representing the genera were fitted onto the ordination using the function envfit. Ordination plots were drawn using the package ggplot2.

## Results

### Taxonomic and functional assignment of fungi

The final dataset based on 39 stands comprised 6,599 OTUs, including 52.3% fungal, 7.59% animal, and 3.6% plant OTUs. The remaining OTUs included algae and protists (30.82%) (*Alveolata, Amoebozoa, Apusozoa, Choanoflagellida, Euglenozoa, Glaucocystoplantae, Heterolobosea, Ichthyosporia, Katablepharidophyta, Rhizaria, Rhodophyta, Stramenopila,* and *Telonemida*) as well as unidentified organisms (5.63%). The fungal dataset included 3,454 OTUs. Overall, 1,296 OTUs were determined to genus and 470 to species level; 2,122 fungal OTUs (61.4%) were identified to fungal functional groups. Approximately half of the OTUs of the overall dataset were assigned to (1) “Other” fungi (animal- and mycoparasites, lichenicolous fungi and pathogens) and (2) organisms of an unidentified fungal group (12.6 and 38.6%, respectively). Major fungal functional guilds (EcM, saprotrophic, and AM fungi) and plant pathogens were analyzed further. All detected fungal reads and Species Hypotheses by the proportion of sequences in both forest and plantation stands are given in Supplementary Table S1.2 and found online under bioproject PRJNA936817 on NCBI.

### Comparison of OTU and sequence proportions

**Figure 1:**
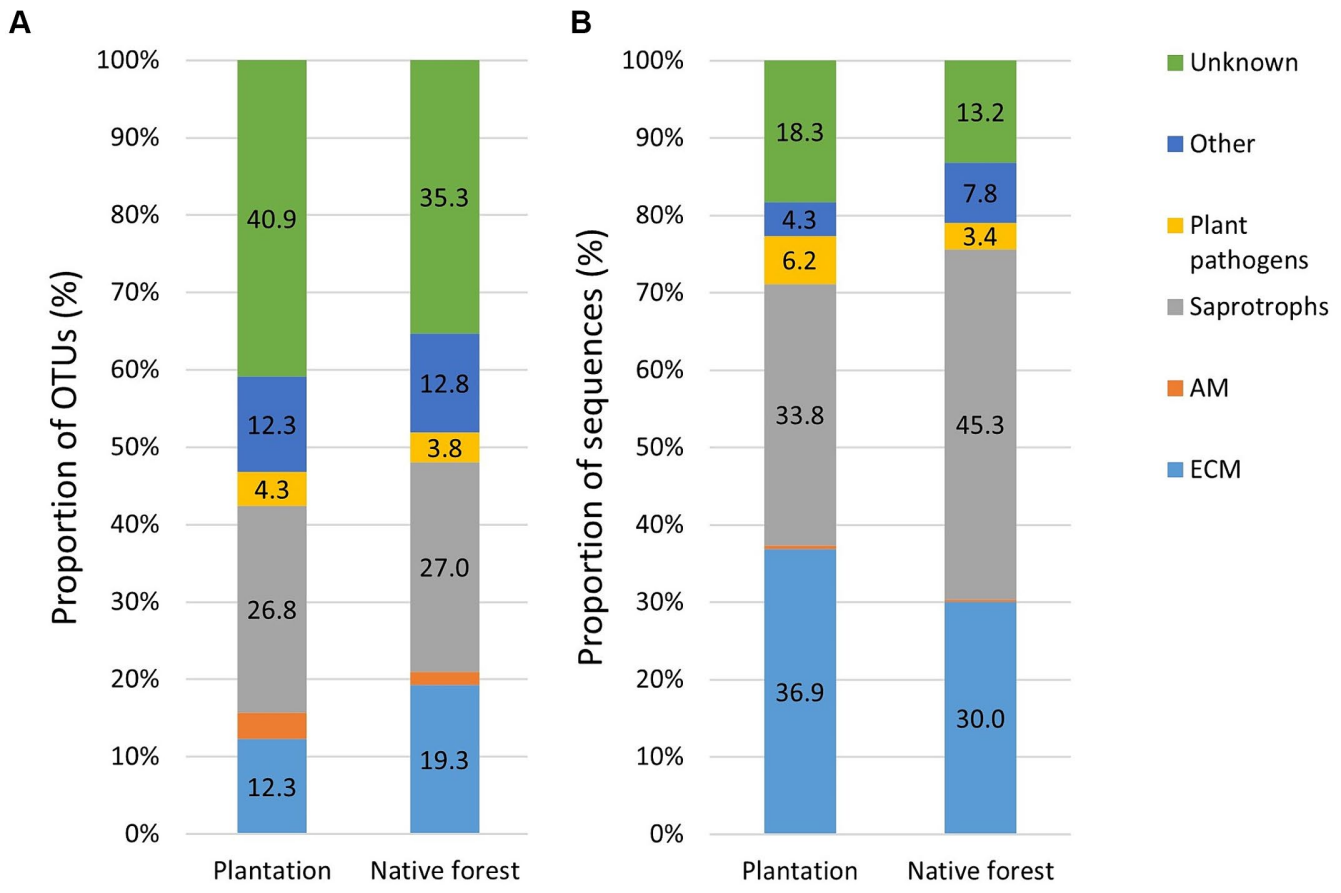
Relative abundances of fungal OTUs **(A)** and sequences **(B)** in hybrid aspen plantations (*n* = 20) and native aspen native forests (*n* = 19). Group “other” includes animal parasites, mycoparasites, pathogens, and lichenicolous fungi. The proportion of AM sequences remained below 1% in both plantation and forest sites.

The proportion of EcM OTUs was higher in native aspen stands compared to hybrid aspen plantations (*R*^2^adj = 0.50; *p* < 0.001), conversely, the proportion of EcM OTUsequences in native aspen stands was lower compared to hybrid aspen plantations (*R*^2^adj = 0.042; *p* = 0.011). OTU and sequence proportion of AM fungi in native aspen stands were lower than in hybrid aspen plantations (*R*^2^adj = 0.199; *p* = 0.003 and *R*^2^adj = 0.045; *p* = 0.104, respectively). OTU and sequence proportion of plant pathogens was significantly higher in plantations compared to native aspen stands (*R*^2^adj = 0.359; *p* < 0.001 and *R*^2^adj = 0.177; *p* = 0.004, respectively). Proportions of saprotroph OTUs and sequences did not differ between forest and plantation (Figures 1A,B).

### Ectomycorrhizal exploration types

Considering all 39 sites, short-contact ectomycorrhizal foraging type comprised 36.1% of OTUs and 41% of all EcM sequences, medium-fringe type 13.5 and 22.7%, contact type 13 and 13.8%, medium-smooth type 10.8 and 7.5%, and long type 1.8 and 0.2%, respectively. We were unable to allocate 24.8% of OTUs and 14.4% of sequences to any EcM exploration type.

**Figure 2:**
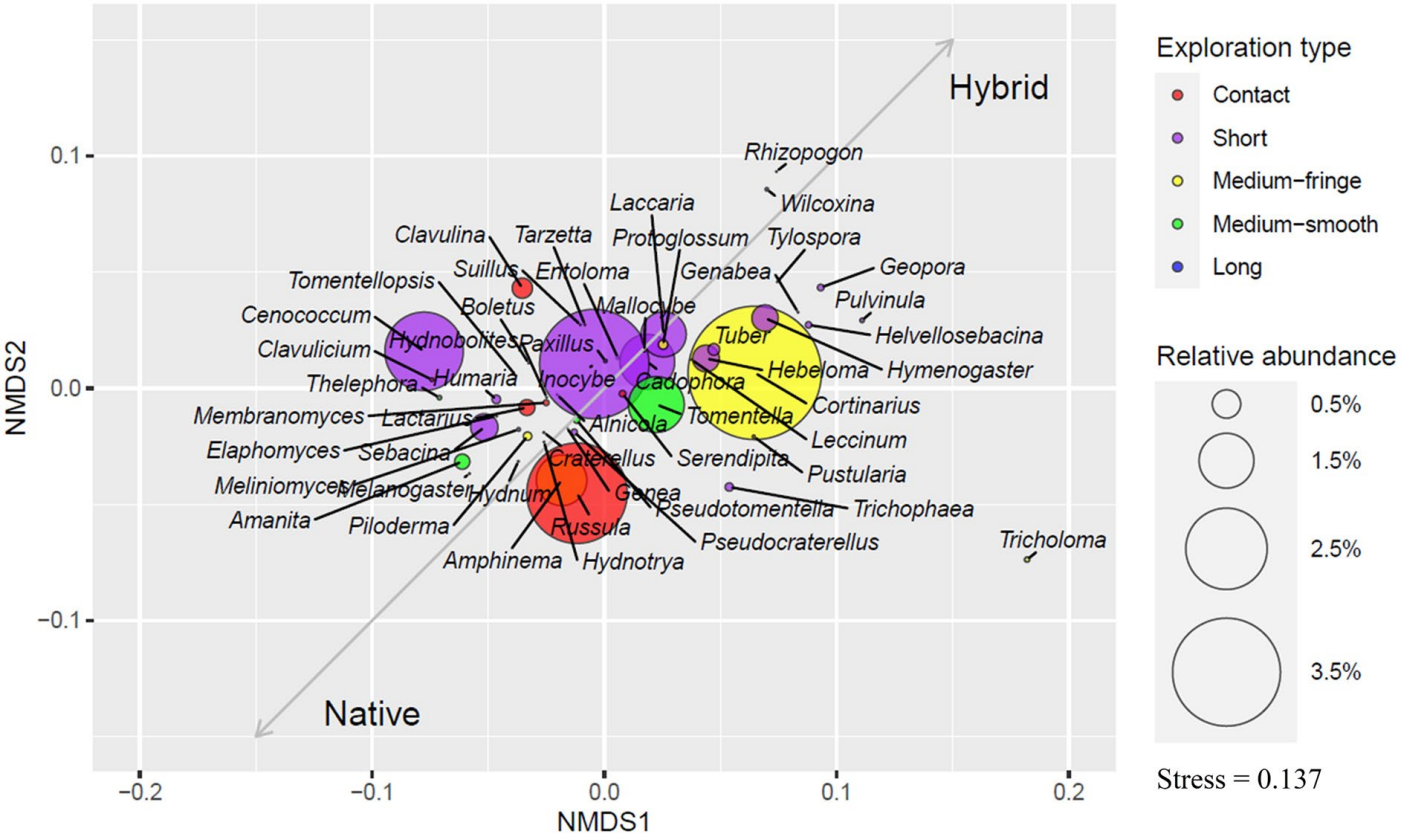
Ordination plot based on the relative abundance of ectomycorrhizal genera. Colors indicate the exploration type of detected EcM genera, and ring sizes show the relative abundance of genera. Vectors indicate which genera are more related to native aspen stands and hybrid aspen plantations.

Short-type genera dominated plantation soils, making up 42% of both OTUs and sequences, followed by medium-fringe type genera that comprised 15.2% of OTUs and 29.2% of sequences, medium-smooth type 8.6 and 6.3%, contact type 6.6 and 7.6% and long exploration type 2.3 and 0.2%, respectively. Undetermined genera OTUs comprised 25.3% and sequences 13.7% in plantations. Short-distance type genera were also most abundant in native aspen soils (31.2%). Contact-type genera OTUs in native aspen stands comprised 59.5% more OTUs and 66% more sequences in native forests compared to plantations. Medium-fringe type OTU abundance was similar between site types but with 54% fewer sequences in native aspen stands. In both OTUs and sequences the percentage of medium-smooth type was 32% higher in native forests. Contrary to our hypothesis, long-distance type out richness were 56% and abundance 24% lower in native forests compared to plantations Levels of undetermined OTUs and sequences were similar in native forests and plantations (23.1 and 15.4%, respectively). An ordination plot illustrating EcM genera exploration types based on their relative abundances is given in Figure 2.

### Genus level comparison

**Figure 3:**
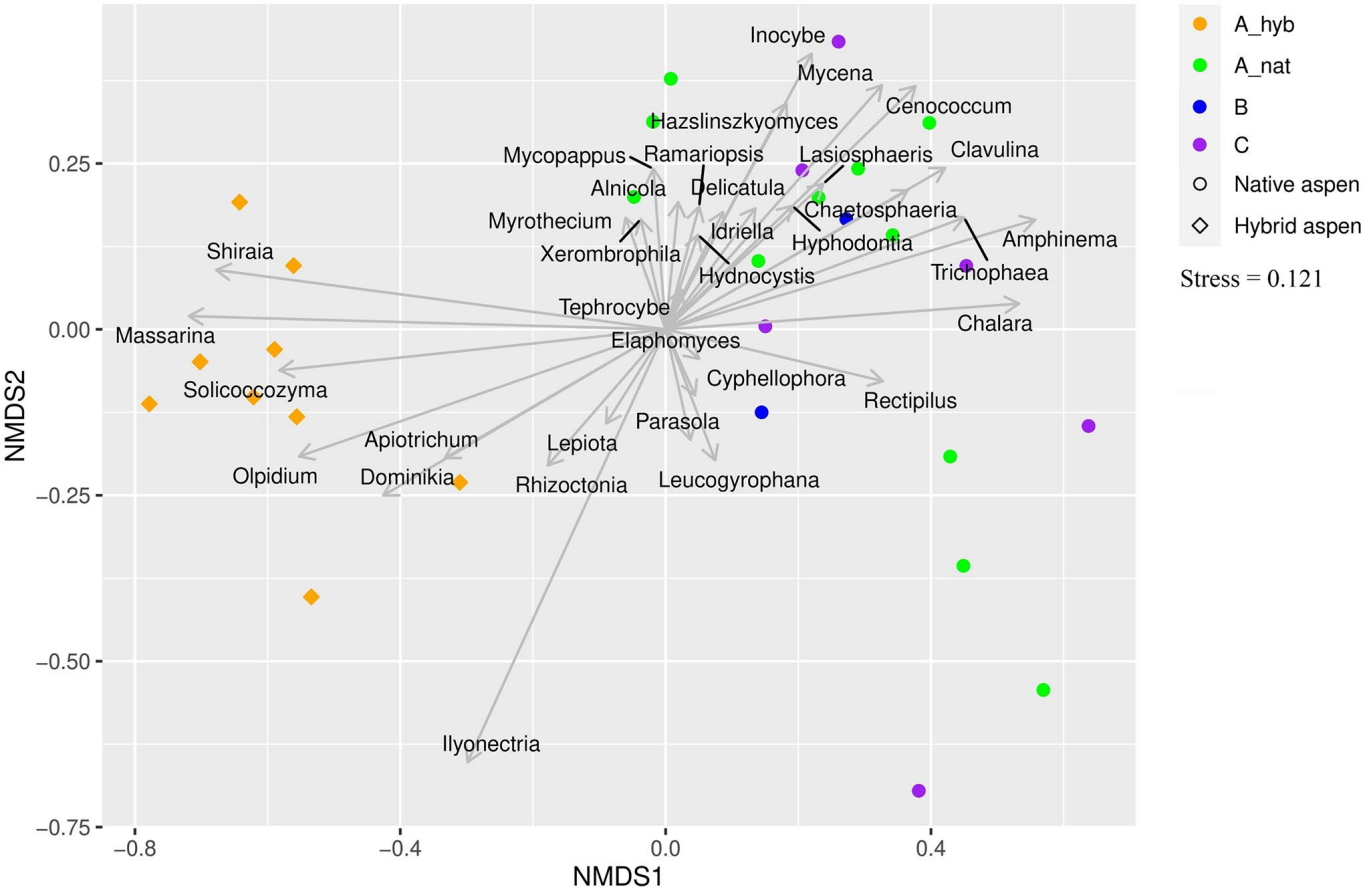
Ordination plot showing associations between fungal genera and plantation and forest age classes. Genera of all fungal groups that were significantly affected by age class are shown on the plot. Symbols represent native aspen stands and hybrid aspen plantations. Colors indicate age classes: A_hyb (hybrid aspen ages 17–18), A_nat (native forest 8–29 y. o), B (native forest 30–55 y. o), and C (native forest 65–131 y. o).

Here, we address which genera of each taxonomic group (EcM, saprotrophs, and plant pathogens) varied significantly (*p* < 0.05) between site and age groups (Figure 3). The statistics and further information are given in Supplementary Table S2 and the ordination plot (Figure 3) displays the patterns in the studied groups.

### Plantation vs. forest

The residual richness of EcM fungi that varied between forest and plantation represented several short-distance exploration type genera (*Mallocybe*, *Pulvinula*, *Genabea*, *Cenococcum*, *Pustularia Genea*, *Capophora*, *Hymenogaster*, *Tuber*, *Sebacina*, *Humaria*, and *Hebeloma*). Late-successional medium-smooth type *Amanita* showed higher richness and relative abundance in native forests as did the late-successional contact-type *Lactarius*. Other contact-type genera such as *Hydnotrya* and *Membranomyces* showed higher richness and relative abundance in native forests. Early-successional short-distance type *Mallocybe* were more abundant and showed higher residual richness in plantations, while early-successional short-distance type *Cenococcum* and medium-smooth type *Thelephora* showed higher residual richness and abundance in native forests (Supplementary Table S2).

Mostly, plant pathogen genera that were significantly different between forest and plantation site types (Supplementary Table S2), showed higher residual richness in plantations. A few genera such as *Ganoderma*, *Heterobasidion*, *Sanchytrium*, and *Venturia* showed higher richness and abundance in forests, but were also present in plantations. Five genera of plant pathogens (*Drechlera, Leptosphaeria, Microdochium, Olpidium*, and *Tilletia*) were absent from forests while *Rhizidium* was absent in plantations. Most plant pathogen genera (*N* = 14) showed higher relative abundance in plantations or were absent in forests.

### Age groups

The residual richness of EcM fungal genera *Alnicola*, *Amphinema*, *Cenococcum*, *Clavulina*, *Elaphomyces*, *Hydnocystis*, and *Trichophaea* responded to age class. Generally, the oldest native forest (C) group showed the highest residual richness, with the exceptions of *Alnicola* and *Clavulina*, which showed the highest richness in the youngest native aspen group (A_nat) and B group, respectively. Relative abundance of EcM genera that were affected by age group included *Amphinema*, *Cortinarius*, *Elaphomyces*, *Hebeloma*, *Hymenogaster*, *Piloderma*, *Pustularia*, and *Russula*, where *Cortinarius* (medium-fringe type), *Hebeloma* (short distance), *Hymenogaster* (short distance), and *Pustularia* (short distance) showed the highest abundance in the hybrid aspen (A_hyb) group. Late-successional contact type *Russula* was most abundant (est.marg.mean = 0.046) in the oldest native forest age class (C), but A_nat (est.marg.mean = 0.045) and B (est.marg.mean = 0.042) group abundances were not significantly different from the oldest group. *Post-hoc* results of all genera that varied significantly between groups are given in Supplementary Table S2 in red text. Most EcM genera differed in relative abundance between A_hyb and A_nat or B and C groups (Supplementary Table S2, see red text). Two short-distance EcM genera, *Alnicola* and *Elaphomyces,* showed significant differences in relative abundance between A_nat and A_hyb vs. B and B vs. C groups. Both relative abundance and residual richness of EcM taxa (see Supplementary Table S2) did not show any differences between the youngest native forest group (A_nat) and the oldest forest group (C), however *Alnicola* and *Elaphomyces* (Supplementary Table S2, Figure 3) differed between both A_nat vs. B and B vs. C groups. Arbuscular mycorrhizal *Glomus* abundance differed between A_hyb vs. A_nat and A_hyb vs. C and was highest in plantations.

Most plant pathogens (*Boeremia*, *Clonostachys*, *Dominikia*, *Fusarium*, *Phoma*, and *Spizellomyces*) were more abundant in plantations. *Mycopappus* and *Venturia* were more abundant in native forest stands than in plantations. The residual richness of plant pathogens was higher in plantations among *Ilyonectria* and *Olpidium*, yet *Mycopappus* had the greatest residual richness in native forests.

Native aspen stands showed a greater EcM residual richness (*R*^2^adj = 0.617; *p* < 0.001, forest OTUs 98 > 60), whereas the richness values of plant pathogens and AM fungi were significantly higher (*R*^2^adj = 0.353; *p* > 0.001; forest OTUs 20 < plantation OTUs 30 and *R*^2^adj = 0.097; *p* = 0.030; forest OTUs 5 < plantation OTUs 11.4, respectively) in hybrid aspen plantations. The total fungal richness and saprotroph richness did not differ between forest and plantation site types (forest OTUs 440 < plantation OTUs 425 and forest OTUs 136 < plantation OTUs 130, respectively). The EcM woody understorey tree species richness (%) did not affect EcM or any other group richness in hybrid aspen or native aspen stands except in overall fungal richness (*R*^2^ = 0.267; *p* = 0.021) of hybrid aspen stands. Similarly, EcM fungi were not affected by EcM host density, although EcM fungal richness showed a weak but not significant correlation with EcM host density (*p* = 0.125, *r* = 0.365). The estimated marginal OTU values of all sites and fungal groups are given in Supplementary Figure S1.

### Fungal richness in hybrid aspen plantations and native aspen stands

The total fungal richness in plantations was negatively associated with woody understorey richness (*R*^2^adj = 0.200; *p* = 0.027). EcM richness was positively affected by the stem wood volume (*R*^2^adj = 0.187; *p* = 0.032), whereas the richness of woody understorey species had a positive effect on EcM fungal abundance (*R*^2^adj = 0.223; *p* = 0.021) and a negative effect on the richness of saprotrophic fungi (*R*^2^adj = 0.225; *p* = 0.020). Significant predictors for plant pathogen abundance were soil P concentration (*R*^2^adj = 0.372; *p* < 0.001; negative effect), the richness of woody understorey species (*R*^2^adj = 0.194; *p* = 0.003; negative effect) and soil C concentration (*R*^2^adj = 0.143; *p* = 0.006; negative effect). For the abundance of AM fungi and saprotrophs and the richness of plant pathogens, there were no significant predictors (Supplementary Table S4).

The richness of all fungi in native aspen sites (Supplementary Table S5) was negatively affected by soil C concentration (*R*^2^adj = 0.194; *p* = 0.016). The richness of EcM fungi was positively affected by N^15^ (*R*^2^adj = 0.296; *p* = 0.006) and negatively affected by the richness of the undergrowth species (*R*^2^adj = 0.234; *p* = 0.011). The abundance of EcM fungi increased along the pH gradient (*R*^2^adj = 0.344; *p* = 0.005; positive effect). The richness of saprotrophic fungi was negatively affected by soil C concentration (*R*^2^adj = 0.248; *p* = 0.017), and the abundance of saprotrophic fungi was negatively affected by soil pH (*R*^2^adj = 0.487; *p* = 0.001). Soil C concentration (*R*^2^adj = 0.337; *p* = 0.005) affected the abundance of AM fungi negatively, although the model did not reveal any significant predictors of their richness nor for the richness of plant pathogens. The abundance of plant pathogens was negatively affected by the soil C/N ratio and stand volume (*R*^2^adj = 0.408; *p* = 0.001 and *R*^2^adj = 0.089; *p* = 0.027, respectively).

Of all observed fungal groups in native aspen stands, only EcM richness was significantly higher in unmanaged stands (*R*^2^adj = 0.283; *p* = 0.016). No fungal group richness was affected by thinning in the hybrid aspen plantations (*p* > 0.05). Similarly, one-way ANOVA showed no significant impact of soil water regime and former land use on any fungal group richness. Additionally, the time since the land had been in agricultural use before hybrid aspen afforestation had no impact on total fungal richness.

**Figure 4:**
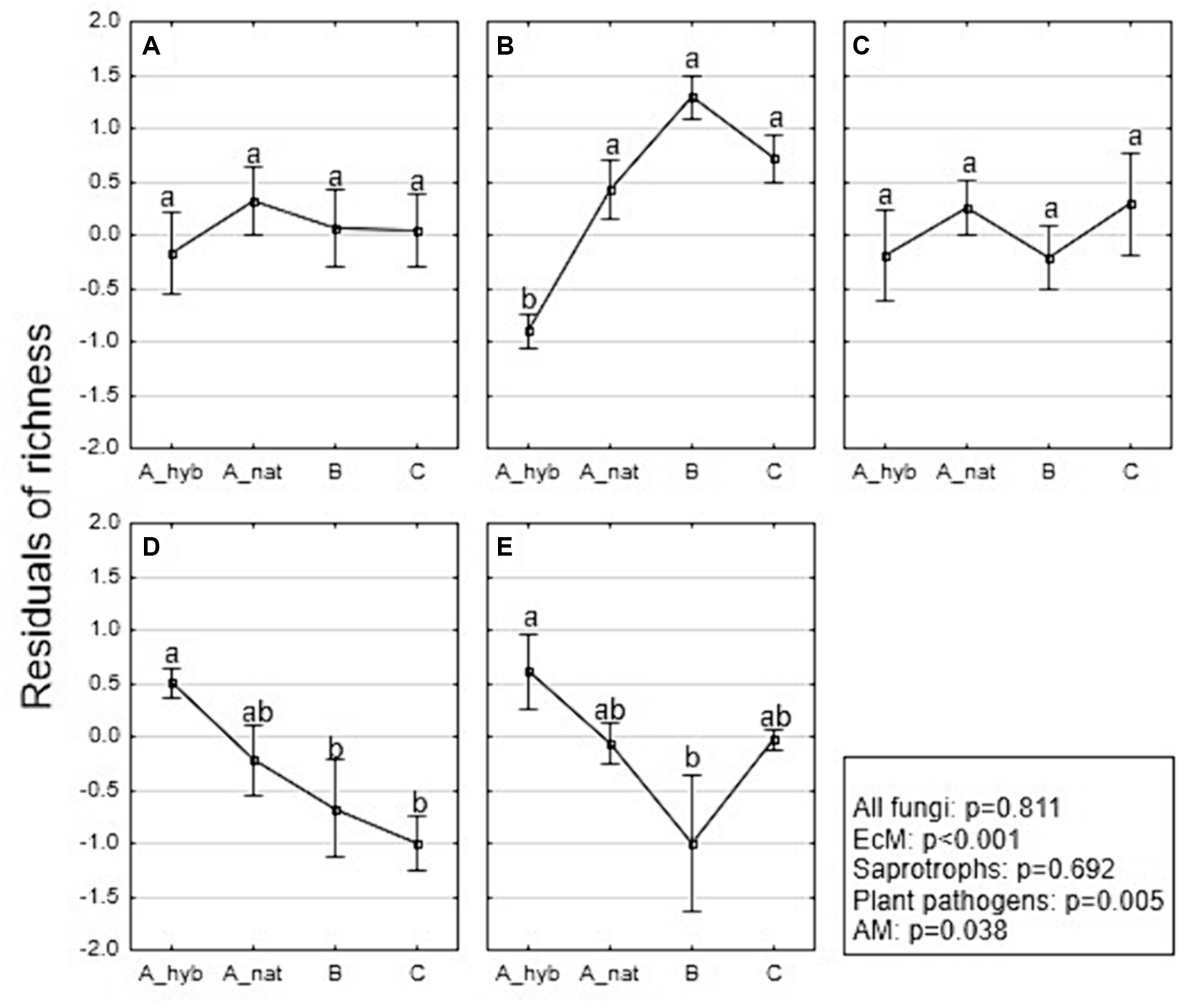
Means of the residual richness of major fungal functional groups [**(A)** all fungi, **(B)** EcM, **(C)** saprotrophic fungi, **(D)** plant pathogens, and **(E)** AM] categorized by age class, where (A_hyb) represents hybrid aspen plantations aged 17–18 years (*n* = 9), (A_nat) refers to native aspen sites aged 8–29 years (*n* = 7), (B) indicates native aspen sites aged 30–55 years (*n* = 6), (C) refers to native aspen sites aged 65–131 years (*n* = 6). Squares represent mean values; whiskers denote standard errors. *p* values are obtained from ANOVA.

Total fungal residual richness showed no significant difference (*p* = 0.811) among stand age classes (Figure 4A). The EcM richness (Figure 4B) was significantly affected by stand age class (*R*^2^adj = 0.682; *p* < 0.001), where A_hyb (plantations 17–18 years) was lower than A_nat (forest stands. 8–29 years) (*p* < 0.001; OTUs 58 vs. 88; see also Supplementary Figure S2), B (forest stands 30–55 years) (*p* < 0.001; OTUs 58 vs. 115), and C (forest stands 65–131 years) (*p* < 0.001; OTUs 58 vs. 93). However, the EcM richness did not differ among forest groups (A_nat, B and C). Age class did not affect the richness of saprotrophic fungi (*p* = 0.692) (Figure 4C). Plant pathogen richness was affected by stand age (*R*^2^adj = 0.333; *p* = 0.005), where the richness of A_hyb was significantly higher than that of the B (*p* = 0.033; OTUs 31 vs. 22) and C (*p* = 0.005; OTUs 31 vs. 15) classes (Figure 4D). The richness of AM fungi (Figure 4E) was significantly (*p* = 0.038) affected by the age class, where the mean richness in hybrid aspen stands (A_hyb) was higher compared to that of other age classes and significantly different from that of class B (*p* = 0.022; OTUs 12 vs. 5). For the native aspen stands, we also performed an analysis for each fungal group, where age was used as a continuous variable. However, the results did not reveal any significant correlation between residual richness and age.

### Fungal community composition

Both PERMANOVA and PCO analyses were applied to the total fungal dataset (*n* = 39, except *n* = 36 for AM communities) and separately for hybrid aspen (*n* = 20) and native aspen (*n* = 19, except *n* = 16 for AM communities) stands to reveal the significant predictors of fungal community composition (Supplementary Table S6). The community composition of all fungi in the overall dataset was explained by stand type (plantation/forest) (18.5%, *p* = 0.001) and soil pH (1.9%, *p* = 0.001) (Supplementary Figure S3). The same predictors were found for EcM fungal communities (Supplementary Figure S4; 22.8%, *p* = 0.001 and 1.3%, *p* = 0.001, respectively). Saprotroph communities (Supplementary Figure S5) were explained by stand type (4.1%, *p* = 0.001), soil C (2.1%, *p* = 0.001), and soil pH (2%, *p* = 0.001). Stand type described 6.5% of the variation in plant pathogen community composition (*p* = 0.001) (Supplementary Figure S6), and soil pH and soil C each explained 1.1% (*p* = 0.002). For AM fungi stand type described 12.2% of the variation in AM community composition (*p* = 0.001) (Supplementary Figure S7).

**Figure 5:**
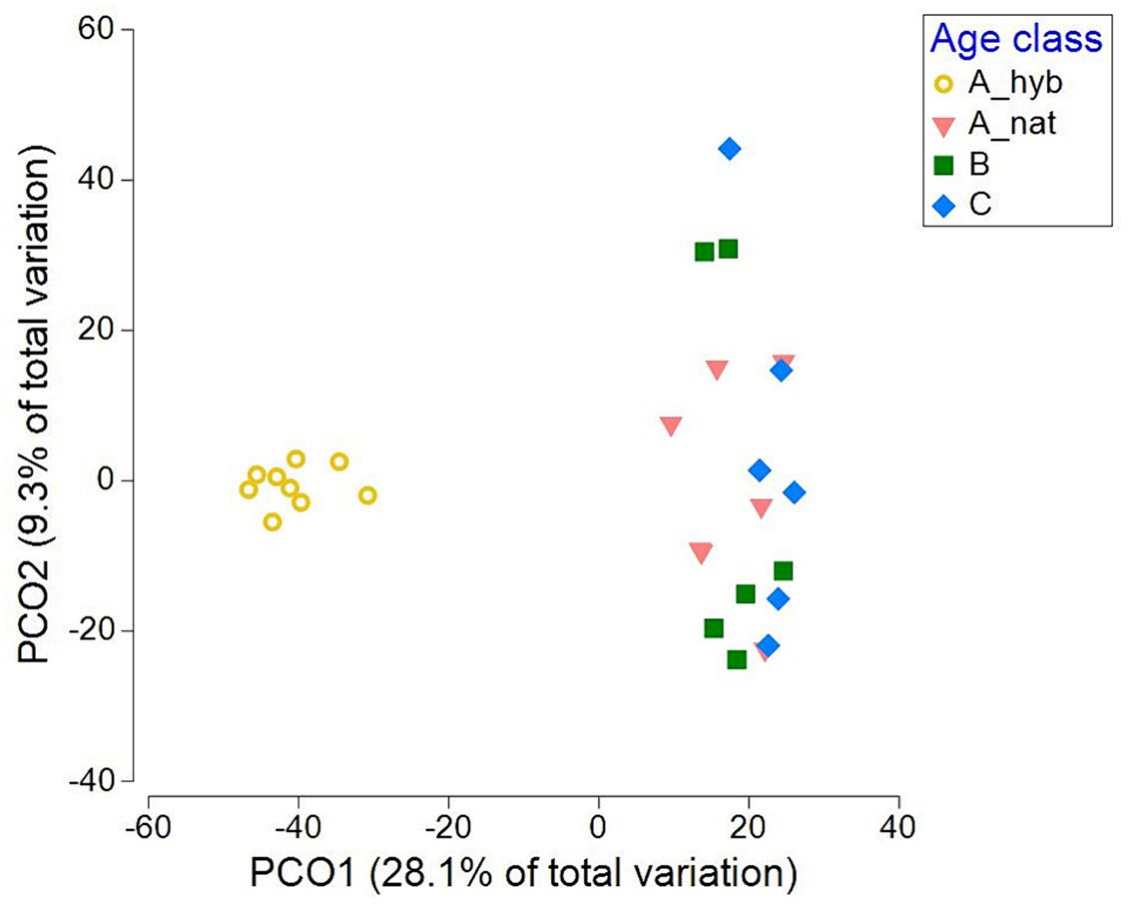
Principal coordinate analysis (PCO) based on the relative abundances of fungal OTUs, describing the variation in EcM community structure, classified by the age classes in selected plantation plots. (A_hyb) = 17–18 years old (*n* = 9) and in forest, (A_nat) = forest sites aged 8–29 years (*n* = 7); (B) = forest sites aged 30–55 years (*n* = 6); and (C) forest sites aged 65–131 years (*n* = 6).

The PCO plots clearly display the significant differences between plantations and forest stands in terms of their communities, with a wider variety of fungal communities of the total, EcM, saprotrophic and pathogenic fungi in *P. tremula* forest stands compared to those in hybrid aspen plantations. The forest plant pathogen and saprotroph communities were slightly more scattered on the PCO plots compared to all fungi and EcM communities, which could explain the weak site effect on pathogen and saprotroph communities (4.1 and 6.5%, respectively) compared to all fungi and EcM (18.5 and 22.8%, respectively). In contrast, for the AM communities stand type described 12.2% of the variation. The AM points on the PCO plot (Supplementary Figure S7) were polarized and the most scattered among the remaining studied fungal groups. The plantation cluster differed strongly (*p* < 0.001) from all forest age classes regarding all fungal groups (Supplementary Figures S8–S11; Figure 5). Only the youngest (A_nat) and the oldest (C) forest EcM communities (Figure 5) showed a marginal significant difference (*p* = 0.065). None of the other fungal guilds showed significant patterns among native forest age classes.

In hybrid aspen plantations, the PERMANOVA results (Supplementary Table S6) showed that soil pH was the most important factor affecting community composition of all fungi, explaining 6% of the variation in fungal community composition (*p* < 0.001), followed by the soil C concentration (2%, *p* = 0.003), the richness of the woody understorey species (1.7%, *p* = 0.005) and the richness of undergrowth vascular plants (1.3%, *p* = 0.017). The variation in EcM community composition was explained by soil pH (7.7%, *p* < 0.001), soil C/N (3%, *p* = 0.003) and soil P concentration (1.5%, *p* = 0.031). Soil pH was the most important factor for the variation in saprotroph community composition, explaining 9.7% of the variation (*p* < 0.001), followed by soil C concentration (1.3%, *p* = 0.020) and soil C/N ratio (1.7%, *p* = 0.008). Soil pH explained 4.5% of the variation in plant pathogen communities (*p* = 0.002), and soil C/N explained 2.9% (*p* = 0.014). The PERMANOVA showed no significant results for AM fungal communities. Overall, thinning, former land use and soil water regime had no significant effects on any of the fungal groups.

In native aspen stands, soil C concentration explained 4% of the variation in total fungal community composition (*p* = 0.003), followed by soil pH (3.2%, *p* = 0.011), stem wood volume (2.3% *p* = 0.013), and richness of the woody understorey species (2.3%, *p* = 0.012). The variation in EcM communities was explained by soil N^15^ (3.1%, *p* = 0.011), richness of the woody understorey species (2.4%, *p* = 0.001), soil pH (2.3%; *p* = 0.019) and soil C concentration (1.9%, *p* = 0.032). Significant predictors of saprotroph communities were soil C concentration (6.2%, *p* = 0.002), soil pH (3.6%, *p* = 0.014), stem wood volume (3%, *p* = 0.010), and richness of the woody understorey species (3%; *p* = 0.014). The variation in plant pathogen communities was explained by soil C concentration (6.9%; *p* = 0.001) and richness of the woody understorey species (3.9%; *p* = 0.020). The AM communities were affected by soil P concentration (8.2%; *p* = 0.014).

## Discussion

Short-rotation forestry with hybrid aspen is an alternative land use for abandoned agricultural lands to increase their economic value and provide climate benefits through CO_2_ fixation (Weih, 2004; Lutter et al., 2021). It also reduces clear-cutting of natural forest stands. However, present knowledge about the biodiversity and succession in hybrid aspen plantations mainly covers vascular plant, bryophyte, and lichen species (Tullus et al., 2015; Randlane et al., 2017), whereas soil fungal communities are poorly studied. To our knowledge, there are no studies on the diversity and composition of soil fungal biota in hybrid aspen plantations planted on previously agricultural lands and comparison of these fungal communities to those of native European aspen forests.

Our results confirmed that EcM dominates over AM fungi after 17–18 years of the establishment of hybrid aspen plantations. We assume that before afforestation, the presence of AM fungi was higher due to the absence of woody plants. Unfortunately, we cannot test this hypothesis, as the first soil samples from the studied sites were collected in 5-year-old plantations in order to determine soil chemical properties. Still, plantation soils showed a significantly greater AM fungal sequence abundance compared to forest sites. This is in agreement with the findings of Lutter et al. (2023), who reported both higher abundance and diversity of AM fungi in birch plantations compared to birch forests.

Plant pathogen abundance was also greater in hybrid aspen plantations, whereas Lutter et al. (2023) found no differences in the abundance of plant pathogens between birch forests and plantations, although the diversity of plant pathogens was significantly higher in birch plantation sites. This could be explained by the hypothesis that former cropland soils host both forest and agricultural land-associated plant pathogens. For example, in the current study grassland-associated *Drechlera* (Smiley et al., 2005; Hu et al., 2018), *Olpidium* (Alexopoulos et al., 1996; Lay et al., 2018) and *Tilletia* (Cannon and Kirk, 2007) were only present in plantations. Mixed stands are also usually considered more resilient to pathogens than monocultures (Piri et al., 1990; Pautasso et al., 2005). Specialist pathogens such as *Heterobasidion* were present in two plantations and seven forest stands. Both plantations and six forest stands contained spruce in their woody understorey species compositions. Furthermore, antagonistic organisms are generally absent in plantations, facilitating the spread of some plant pathogens (Swedjemark and Stenlid, 1993). *Ganoderma* spp., potential pathogens usually associated with older trees, were present in all studied sites. The rare saprotrophic *Ganoderma lucidum,* usually associated with older trees, was present in three forest stands. However, we found the species from 14-, 40-, to 131-year-old forests. Some aspen clones are more resistant to bacterial (Nesme et al., 1994) and fungal pathogens (Kasanen et al., 2002) (for example: *Xanthomonas populi* and *Neofabrea Populi*, respectively) than others. An interesting study suggested a “safe” number of different hybrid aspen clones when practicing plantation forestry to prevent pest damage ranged from 30 to 40 (Roberds and Bishir, 1997). In our studied hybrid aspen plantations, approximately 15 different clones were randomly planted across the site to avoid large monoclonal tree groups. Hybrid aspen is also an exotic species, and as the plantations are established with a limited number of clones they are considered vulnerable to pest and disease outbreaks (Tullus et al., 2012; Wu, 2019; Lutter et al., 2019). Some pathogens that pose a threat to aspen are undetectable from soil samples and our study does not reflect the entire plant pathogen picture in hybrid aspen plantations. For instance, serious canker-causing *Entoleuca mammata* is detectable only from symptomatic aspen stem or canker samples (Anderson, 2005; Lutter et al., 2019).

Saprotroph richness and relative abundance of sequences was similar in aspen plantations and forests in this study (Figures 1A,B). This trend is in contrast to that found in birch plantations and forests where a significantly higher relative abundance of saprotrophic fungi was found in the forest sites, although the OTU diversity was similar (Lutter et al., 2023). This suggests that plantation soil saprotroph communities are dependent on host plant species.

It is important to note that the detected EcM taxa from soil samples do not necessarily form a symbiosis with aspen. In order to determine such a symbiosis root-tip analyses would need to be done (Szuba, 2015). However, our main purpose was to describe soil fungal communities in plantations and native aspen forests more broadly. We expected EcM diversity to be higher in forest sites, a hypothesis which was partly confirmed. While the EcM sequence abundance was higher in plantations, the number of EcM OTUs in forest sites was significantly higher compared to that in plantations (Figures 1A,B). A similar trend has also been observed for birch plantations and forests (Lutter et al., 2023). However, we found no differences between plantations and native forests in terms of residual richness of EcM fungi. Genera-wise, we found that late-successional *Amanita* species occurred in only one plantation whereas they were present in 15 native forest sites. Late-successional *Lactarius* species abundance and residual richness in plantations were significantly lower than that in native forests. As we covered only significantly different genera between different levels (age classes and site), the late-successional taxa we could determine were rather scarce and clear conclusions based on so few genera cannot be stated. However, based on the difference between the abundances of *Amanita* and *Lactarius*, late-successional taxa were more abundant in native forests and this is consistent with earlier works (Cripps and Miller, 1993). Many short-distance type taxa of EcM fungi were significantly more abundant (nine genera) and showed higher residual richness (seven genera) in plantations than in native forests (Supplementary Table S2). Short-type EcM genera such as *Cenococcum*, *Genea*, *Humaria*, *Hymenogaster*, *Sebacina*, and *Thelephora* also dominated in forest soils (Figure 2).

The richness of the woody understorey species correlated with the richness of all fungi and saprotrophs as well as the abundance of plant pathogens and EcM fungi in plantations but had little effect on EcM fungi in native forests. On the contrary, community compositions of all fungal guilds, except AM fungi, responded to woody understorey species when all sites (*n* = 39) were analyzed together, whereas it did not have an effect on any fungal guild in plantations. Forest stand richness was mainly affected by soil properties, whereas the hybrid aspen stand richness was mostly explained by woody understorey species richness. A similar study investigating native birch stands and plantations found contrary patterns (Lutter et al., 2023); the soil properties only slightly influenced the fungal richness, whereas land use history and surrounding forest area were the most important predictors. The present study found no effect of the nearest forest on plantation fungal richness. A previous study on Norway spruce stands (former agricultural land and forest sites) found that differences among soil fungal communities were mainly influenced by soil pH and sampling site (Klavina et al., 2022). Spatial distribution analysis (Ramsfield et al., 2020) showed that undisturbed forests provide a source of EcM fungi, allowing them to disperse into reclaimed areas, and that overall fungal richness is even higher than that of the adjacent undisturbed forest within 10–20 m from the forest edge. Although spores could theoretically spread hundreds of kilometers, they usually fall within 1 m of the fruitbody (Galante et al., 2011), and only 1% of spores spread further than this (Golan and Pringle, 2017). A study in short-rotation willow coppice showed a poor relation between EcM abundance and diversity and their neighboring native sites, which was explained by insufficient spore dispersal (Hrynkiewicz et al., 2012). Similar to the fungal richness in hybrid aspen plantations, distance from the forest as a continuous factor did not explain fungal communities or richness. When three distance groups were analyzed separately (35–125, 130–180, and 200–670 m), we still could not detect any similarities in fungal community composition within the groups or a significant impact on any fungal community composition (Supplementary Table S6). This leads us to infer that colonization by some of these taxa from nearby forestsmay take longer than one short forest rotation.

A study in Germany with mycorrhizal deciduous tree species, conducted by Ferlian et al. (2021), showed that EcM fungal richness increased with the increasing diversity of EcM trees but decreased when AM and EcM trees were mixed on the same plot. In our study, we found no significant increase in EcM fungal richness with increasing EcM plant richness. However, EcM richness in plantations increased significantly with increasing stem wood volume. In contrast, AM richness decreased significantly with increasing stand volume. This could be linked to the higher volume of EcM tree species, which may reduce the success of AM fungi due EcM fungi being more dominant and the loss of potential mutualistic plants after afforestation or other land use change. Otsing et al. (2021) showed that tree species richness had neither a significant effect on EcM fungal richness nor community composition, but tree species identity was a significant determinant of EcM community composition. Similarly, Tedersoo and Lindahl (2016) described single tree species as a stronger factor of soil biota than tree species diversity. Our results are partly consistent with the two previous studies, since we found no significant impact of woody understorey species richness and woody EcM understorey species richness on fungal richness in native aspen sites. Unexpectedly, we found a negative correlation between woody understorey richness and EcM richness in native aspen stands and between the richness of saprotrophic and total fungi in hybrid aspen plantations. A negative correlation could be explained by species compositions of woody understorey, as non-EcM understorey species were present in the study sites. However, the residual richness and relative abundance of AM fungi had no correlation with the proportion of EcM understorey woody species. Equally important could be the density or abundance of both EcM and non-EcM woody understorey species. However, no measurement of the abundance of woody species, other than basal area calculation, was done in this study. Regarding fungal abundance, plant pathogens had a significant negative relation with woody understorey richness. This could point to the significant impact of herbaceous or crop-related pathogens in plantations on former agricultural land. Both herbaceous and woody-plant-related pathogens were present in plantations and forests (for example *Heterobasidion* and *Neofabrea*) but rarely in plantations. On the contrary, EcM fungal abundance showed a significant positive correlation with woody understorey richness as EcM fungi may suppress soil-borne pathogenic fungi by ensheathing feeder roots and acidifying soil (Tedersoo et al., 2020). The abundances of some taxa in both fungal groups are dependent on the density of conspecific tree individuals but are affected differently. Nonetheless, *Populus* spp. is known to associate with fungi from many genera (Lodge and Wentworth, 1990) and *Populus tremula* could host over 200 ectomycorrhizal species (Bahram et al., 2011a). While pathogen abundance in roots (although not soil) increases with tree density, EcM abundance is not affected by host density, as reported by Liang et al. (2021). Based on the basal areas, EcM fungi were not affected by EcM host density in this study.

We categorized the sites into four age classes: hybrid aspen sites aged 17–18 years and native aspen stand aged 8–29, 30–55, and 65–131 years. The overall fungal richness did not differ among the age groups (Figure 4A). The EcM richness was the highest in native aspen stands aged 30–55 years but did not differ significantly from that of the stands aged 8–29 and 65–131 years. Not surprisingly, the hybrid aspen group had the lowest richness of EcM fungi. Late-successional *Russula* abundance was similar between all forest age groups. The age groups of AM richness were inversely proportional to the EcM results, where the highest richness was found in the hybrid aspen plantations and the lowest in the native aspen stands aged 30–55 years. Generally, in northern Europe AM taxa are more diverse and abundant in grass- and cropland soils than in forest soils and are suppressed in hemiboreal forests due to EcM plant abundance (Ferlian et al., 2021). However, the result could be more related to the legacy of the former agricultural land-associated biota as during the transition period arbuscular mycorrhizal species are still present (Lutter et al., 2023). Plant pathogen richness was lowest in the native aspen stand aged 65–131 years and highest in the youngest stand (8–29 years). Somewhat unexpectedly, saprotroph richness did not differ significantly among the four age groups. The selected hybrid aspen plantations showed richness values similar to those of native aspen stands aged 30–55 years. It has been previously claimed that wood-inhabiting saprotroph richness and communities are not affected by stand age but rather by decay stage and trunk volume (Runnel and Lõhmus, 2017; Runnel et al., 2021). However, decaying wood volume is usually related to stand age. Soil saprotrophs can remain unaltered by age, as shown in this investigation.

Our models showed that nitrogen isotope levels were the strongest predictor for both residual richness and community compositions of EcM fungi in native aspen stands. Usually, the availability of N determines the richness of EcM fungi. For example, Awad et al. (2019) found that resources from soil and roots, such as N and C concentrations or C/N ratios, had the highest impact on saprotrophic and EcM fungal biomass. Likulunga et al. (2021) found C/N ratio and pH were the main drivers of soil fungal communities. Other studies have shown a significant negative correlation between fungal species richness and N mineralization, indicating that an increase in mineral N is associated with a decline in saprotrophic and EcM richness (Buée et al., 2011; Maaroufi et al., 2019). All told, interpreting soil nitrogen isotope levels to describe N availability is challenging due to the many factors that influence its value. But overall, it shows the turnover of the nitrogen cycle: higher levels are more prone to N losses and lower values refer to more closed N systems where soil holds the nitrogen more (Baumgartner et al., 2021). Roughly, ^15^N can be used as a measure describing nitrogen availability for plants.

Community analyses with all studied sites showed a highly significant difference between fungal communities in hybrid aspen plantations and native aspen forest stands (Supplementary Figures S3–S7). A similar outcome was found in a study comparing first-generation silver birch plantations on former agricultural lands with birch stands on native forest land (Lutter et al., 2023) and in studies that compared native forest fungal communities with those of plantation forests (Kasel et al., 2008; Moora et al., 2014). In the current study, the scattered symbols of forest sites (Supplementary Figures S3–S7) on the plots also indicated a larger variability among fungal communities in the forest environment, whereas the hybrid aspen fungal communities were more similar among plantations. This indicates community homogenization in hybrid aspen plantations, at least when the first rotation of short-rotation hybrid aspen forestry is considered.

The plantation and natural forest datasets, when analyzed separately, showed significant relations between edaphic factors and soil fungal communities, whereas former land use, soil water regime (in plantations), thinning, or age class (in forests) had no impact on fungal communities. Hrynkiewicz et al. (2012) found the higher proportion of colonization of EcM fungi of *Salix* sp. in natural forests compared to short rotation coppice was explained by soil water content and pH. Here, we showed that soil pH had a significant impact on the fungal communities of both plantations and natural forests, except for the plant pathogen and AM fungal communities in native aspen stands, a fact known from several studies (Genevieve et al., 2019; Tedersoo et al., 2020, Likulunga et al., 2021; Klavina et al., 2022). Other studies have revealed that AM communities are strongly affected by soil pH (Dumbrell et al., 2010); whereas their relative abundances are positively influenced by soil N content and negatively by soil P (Koorem et al., 2014). We expected to see a higher fungal richness in natural forest stands than in plantations. However, this hypothesis was only partially confirmed, as the richness was only significantly higher for EcM fungi in native aspen forest soils compared to hybrid aspen plantations.

Surprisingly, aspen-related species and the valued edible mushroom *Leccinum aurantiacum* were only detected in two plantation sites. We believe that this is an underestimate and does not reflect the presence and abundance of this fungus based on personal observations of sporocarps in the studied plantations. Such a result could be related to sampling time, i.e., *Leccinium auratiacum* external mycelia was suppressed by other taxa that were more active at the sampling time. A study in Austria found no *Populus tremula-*associated EcM taxa that were significantly associated with summer or autumn conditions (Krpata et al., 2008). Yet many studies have found significance of seasonal factors on fungal relative abundance (Zhao et al., 2023) and community compositions (Vargas-Gastélum et al., 2015; Beidler et al., 2023). Others have suggested that host-tree characteristics have more impact than the seasonal factor alone in describing changes in fungal communities (Park et al., 2020), thus the seasonal factor should be considered in interaction with tree or site characteristics. Our soil samples were collected in the middle of the growing season (June–July). Host generalist taxa may be less sensitive to environmental changes than host-specialist fungi (Bahram et al., 2011b).

## Conclusion

Our study demonstrates that fungal community compositions of hybrid aspen plantations on former agricultural soils and native European aspen-dominated forests on forest land are distinctly different. Furthermore, the plantation communities were more homogeneous among sample plots compared to those of natural forest stands. Overall, edaphic factors and woody understorey species richness are more important in explaining the fungal diversity and community compositions in hybrid aspen plantations than factors such as recent thinning, former land use (grass- or cropland), time since former agricultural land use before afforestation, and soil water regime. The conversion of abandoned agricultural land into short-rotation forest plantations can be regarded as a promising restoration method for abandoned lands not only for woody biomass production but also to increase biodiversity and host common forest soil species. Since there were numerous fungal genera whose richness or abundance did not differ between natural forests and plantations, we conclude that first-rotation hybrid aspen plantations show great potential to host fungal taxa of natural forest soils. The establishment of hybrid aspen plantations on abandoned agricultural lands can reduce felling in native European aspen forest ecosystems, which are considered to host a diverse richness of other species. Apart from intensive biomass production, plantation forestry (afforested agricultural or abandoned land) supports the maintenance of biodiversity while forming resilient and productive ecosystems in the context of a changing climate. Further investigations could explain how successive rotations alter the fungal richness and the potential of hosting species throughout the rotation cycle in forest plantations.

## Data availability statement

The datasets presented in this study can be found in online repositories. The names of the repository/repositories and accession number(s) can be found in the article/Supplementary material.

## Author contributions

ER: Conceptualization, Data curation, Formal analysis, Investigation, Methodology, Project administration, Resources, Validation, Visualization, Writing – original draft, Writing – review & editing. RL: Data curation, Investigation, Resources, Writing – review & editing. TR: Conceptualization, Formal analysis, Investigation, Methodology, Visualization, Writing – original draft, Writing – review & editing. TT: Data curation, Resources, Supervision, Validation, Writing – original draft, Writing – review & editing. AT: Supervision, Validation, Writing – review & editing. LT: Data curation, Resources, Supervision, Validation, Writing – review & editing. RD: Data curation, Resources, Supervision, Validation, Writing – review & editing. HT: Conceptualization, Data curation, Resources, Supervision, Validation, Writing – review & editing, Funding acquisition.

## Footnotes

^1^www.jarvselja.ee

^2^www.primer-e.htm

## Supplementary material

Supplementary Table S1 Site and soil characteristics (Supplementary Table S1). Detected species hypotheses and their proportions in forest and plantation (Supplementary Table S1.2).

Supplementary Table S2 Variations in fungal genera in forest and plantation.

Supplementary Table S3 List of sources determining the woody undergrowth species symbiotic status (Supplementary Table S3).

Supplementary Table S4 Best predictors of soil fungal richness and relative abundance in major fungal trophic groups of hybrid aspen plantations (Supplementary Table S4) and native European aspen stands (Supplementary Table S5).

Supplementary Table S5 Permanova and Permdisp main and pairwise results.
