# Supplementary material for: Soil mycobiomes in native European aspen forests and hybrid aspen plantations have a similar fungal richness but different compositions, mainly driven by edaphic and floristic factors
Source: Front Microbiol. 2024 May 7;15:1372938. doi: 10.3389/fmicb.2024.1372938 (PMC11106484; doi:10.3389/fmicb.2024.1372938)
Supplement: Supplementary file 6 [file Presentation_1.pdf]

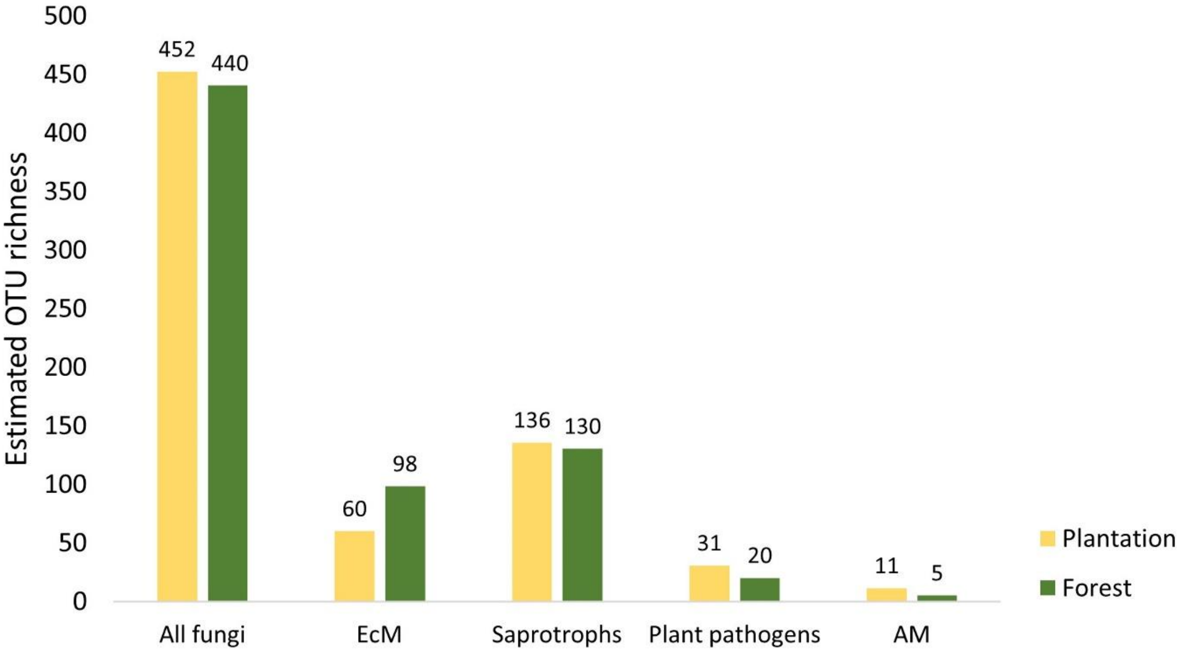

Figure S1. Estimated marginal OTU values of all sites and fungal guilds, categorized by plantation (yellow) and forest (green) sites

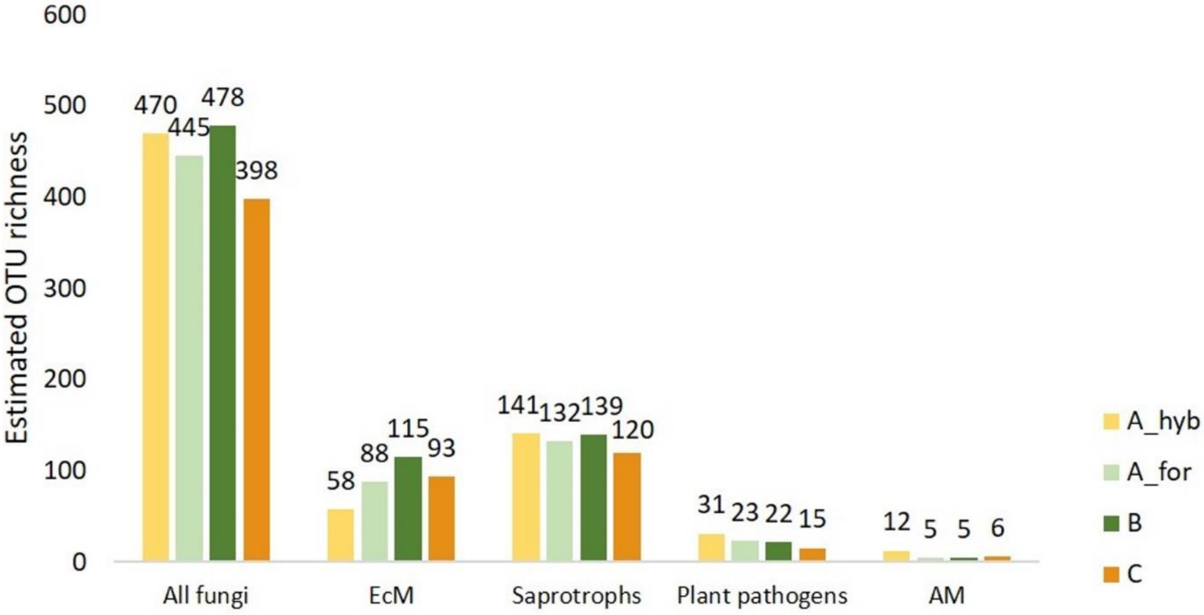

Figure S2. Estimated marginal OTU values of studied fungal guilds in selected hybrid aspen plantations and all European aspen stands, categorized by age classes, where **(A_hyb)** represents the hybrid aspen plantations aged 17–18 years (n = 9), **(A_nat)** refers to European aspen sites aged

8–29 years (n = 7), **(B)** indicates European aspen sites aged 30–55 years (n = 6), **(C)** refers to European aspen sites aged 65–131 years (n = 6)

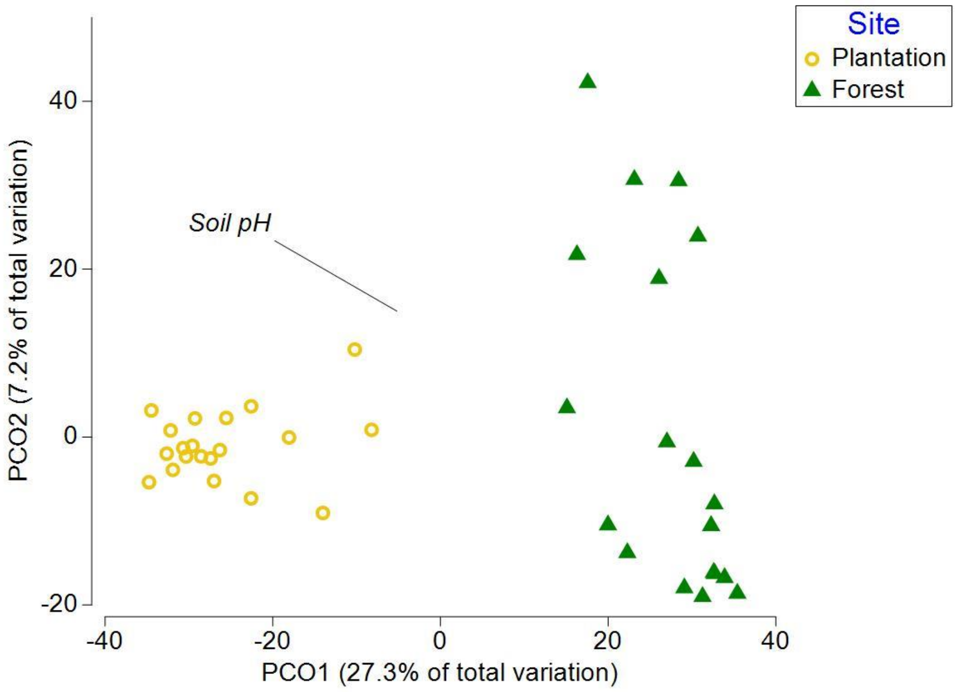

Figure S3. Principal coordinate analysis (PCO) based on the relative abundance of all fungal OTUs, describing the variation in fungal community structure in hybrid aspen plantation (n=20) and forest (n=19) sites. Vector represent the direction of the impact of soil pH concentration.

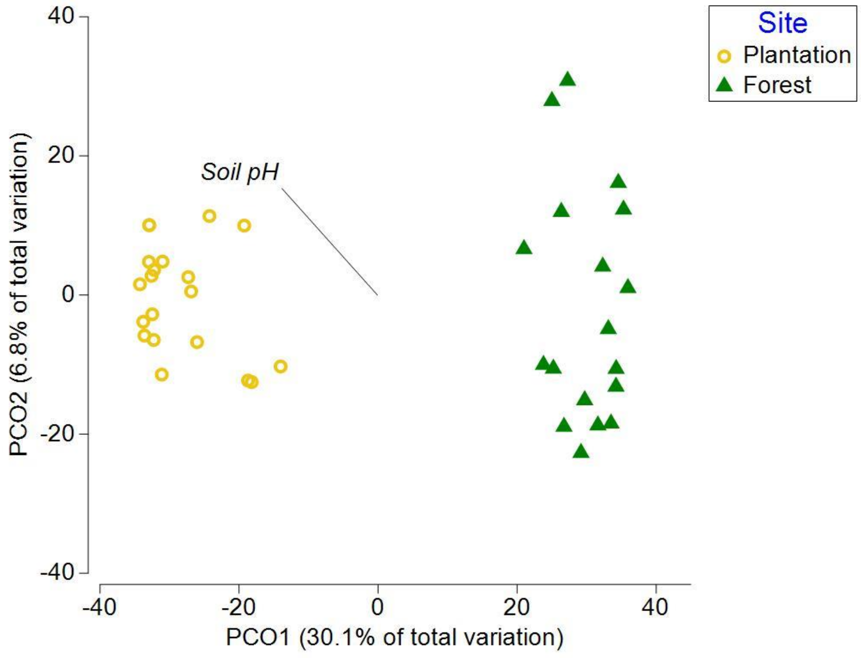

Figure S4. Principal coordinate analysis (PCO) based on the relative abundance of fungal OTUs, describing the variation in EcM fungal community structure in hybrid aspen plantation (n=20) and forest (n=19) sites. Vector represent the direction of the impact of soil pH concentration.

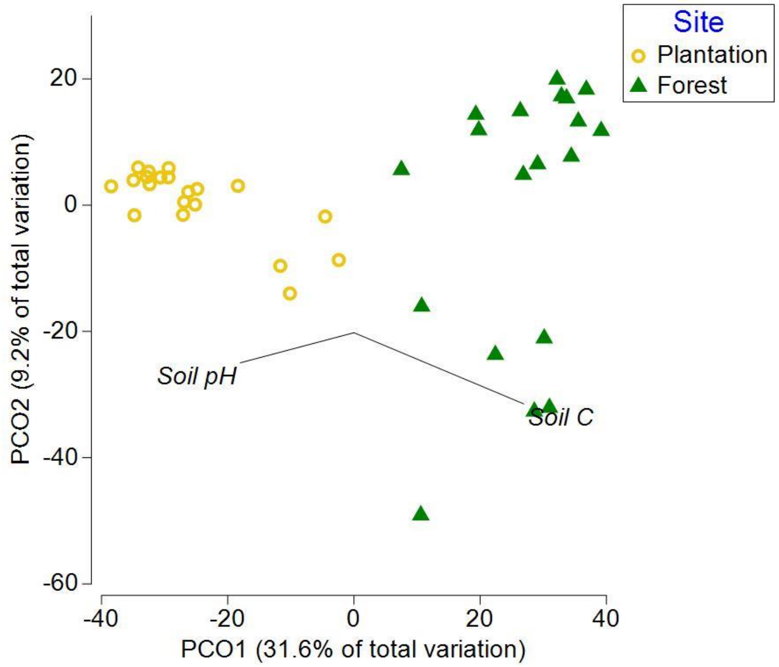

Figure S5. Principal coordinate analysis (PCO) based on the relative abundance of fungal OTUs, describing the variation in saprotroph community structure in hybrid aspen plantation (n=20) and

forest (n=19) sites. Vectors represent the direction of the impact of soil C and soil pH concentration.

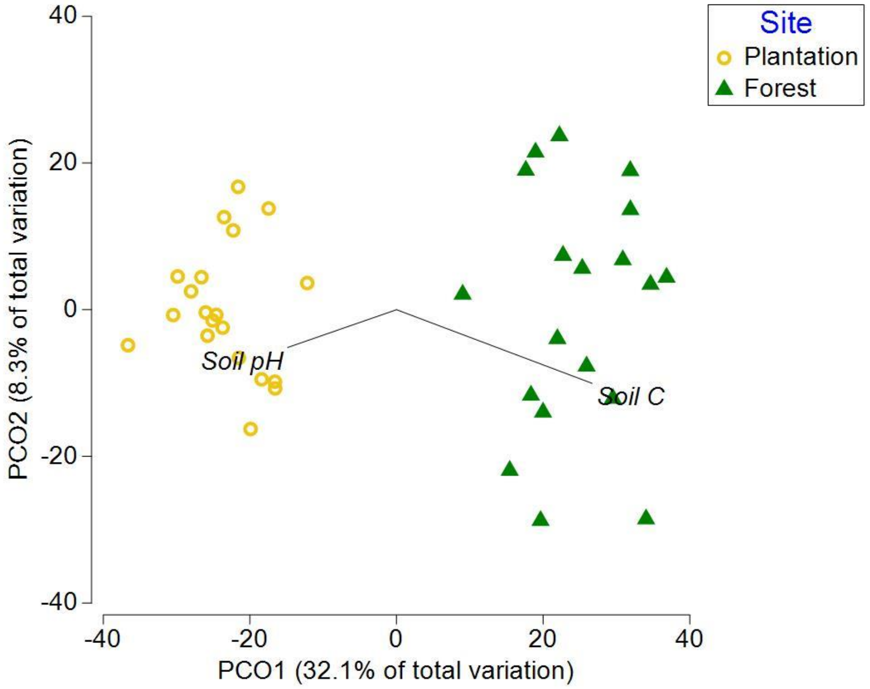

Figure S6. Principal coordinate analysis (PCO) based on the relative abundance of fungal OTUs, describing the variation in plant pathogen community structure in hybrid aspen plantation (n=20) and forest (n=19) sites. Vectors represent the direction of the impact of soil C and soil pH concentration.

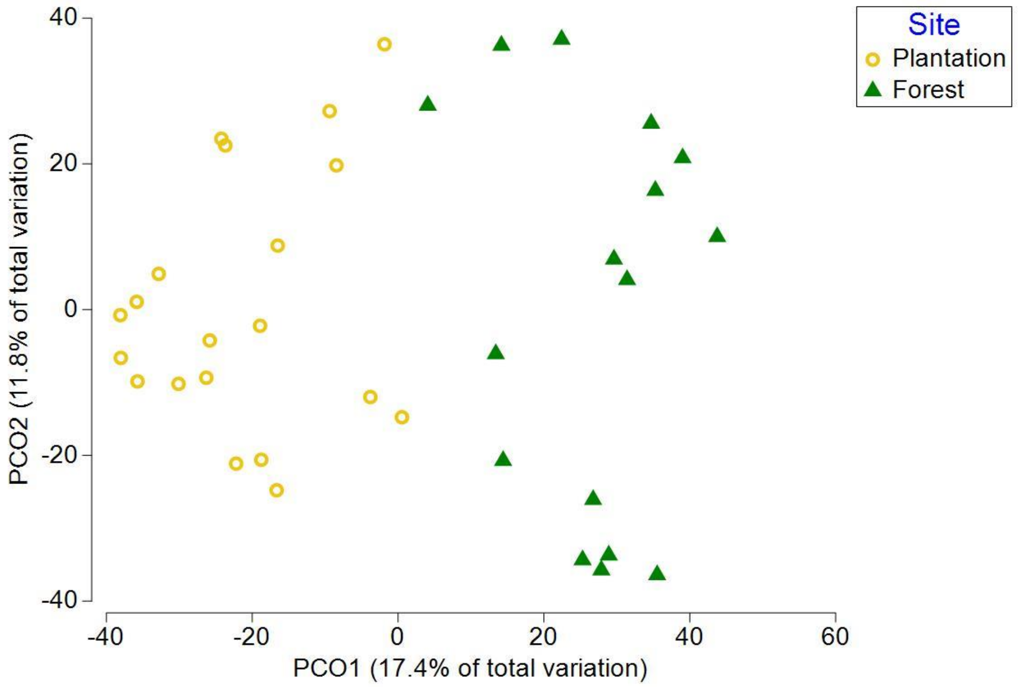

Figure S7. Principal coordinate analysis (PCO) based on the relative abundance of fungal OTUs, describing the variation in AM community structure in hybrid aspen plantation (n=20) and forest (n=16) sites.

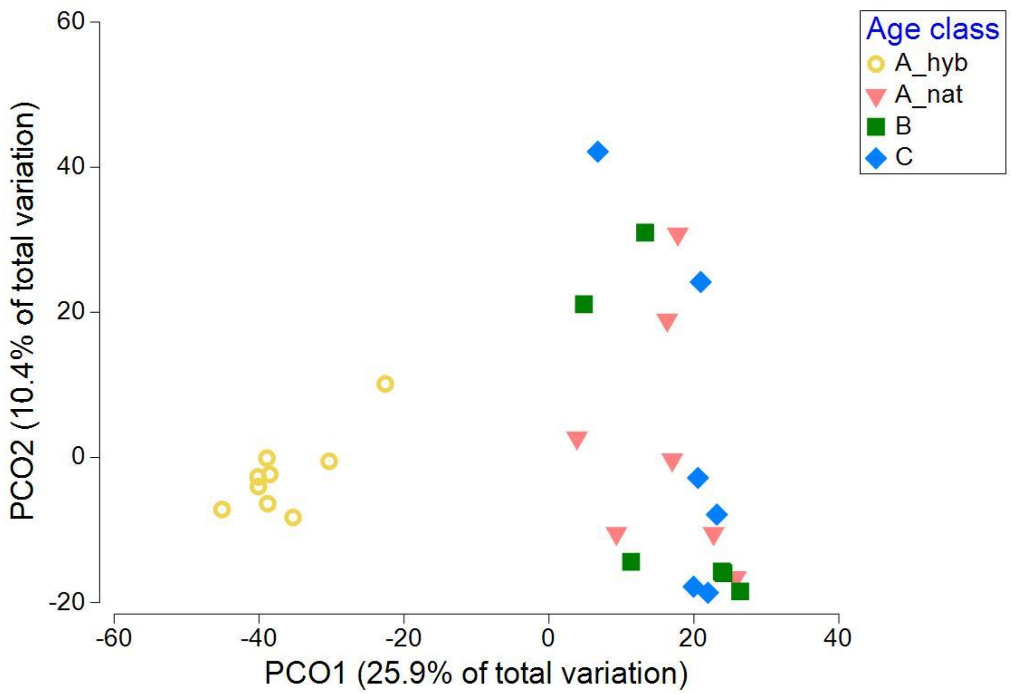

Figure S8. Principal coordinate analysis (PCO) based on the relative abundance of fungal OTUs, describing the variation in total fungal community structure, classified by the age classes in selected plantation plots (A_hyb=17-18 year old, n=9) and in forest (A_nat= forest sites in age 8-29 (n=7); B= forest sites in age 30-55 (n=6) and C – European aspen sites in age 65-131 (n= 6).

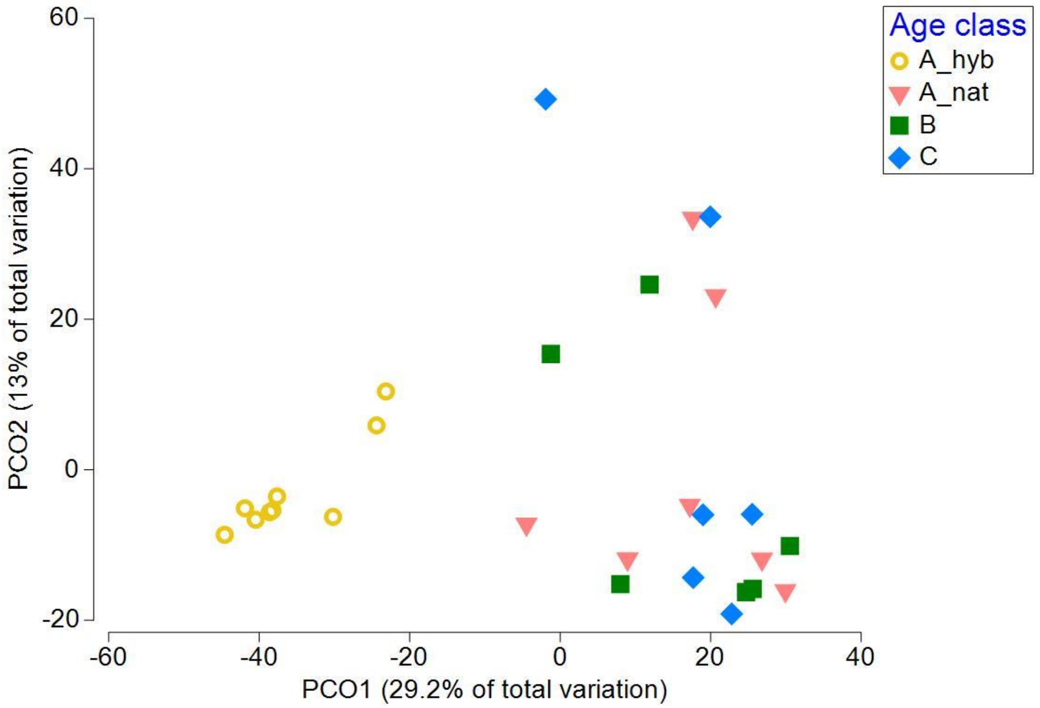

Figure S9. Principal coordinate analysis (PCO) based on the relative abundance of fungal OTUs, describing the variation in saprotrophs community structure, classified by the age classes in selected plantation plots (A_hyb=17-18 year-old, n=9) and in forest (A_nat= forest sites in age 8-29 (n=7); B= forest sites in age 30-55 (n=6) and C – European aspen sites in age 65-131 (n= 6).

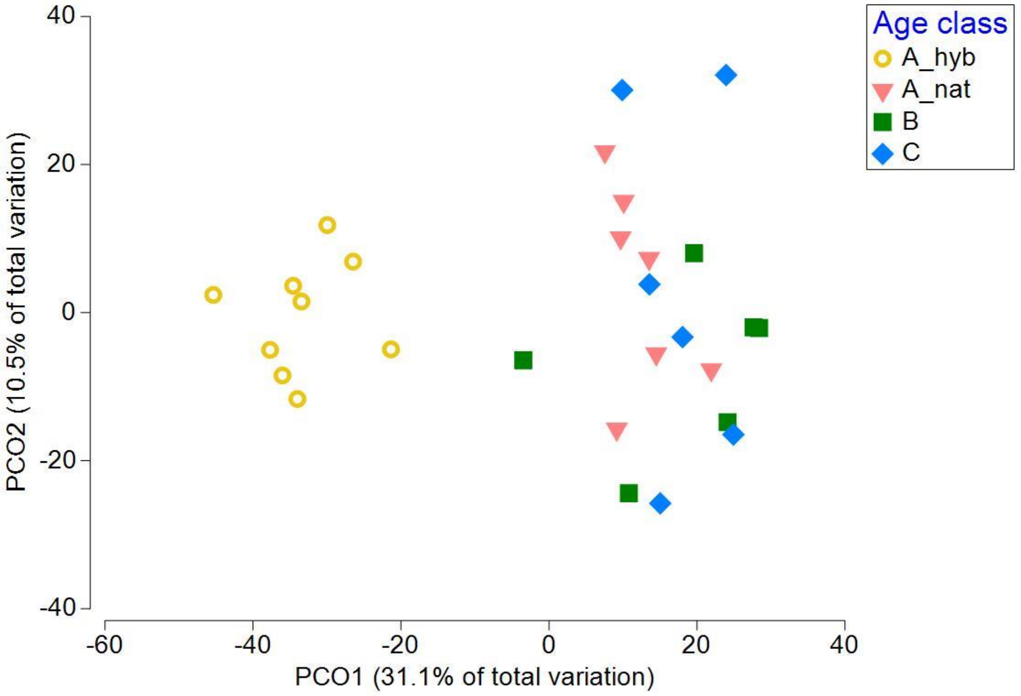

Figure S10. Principal coordinate analysis (PCO) based on the relative abundance of fungal OTUs, describing the variation in plant pathogen community structure, classified by the age classes in selected plantation plots (A_hyb=17-18 year old, n=9) and in forest (A_nat= forest sites in age 8-29 (n=7); B= forest sites in age 30-55 (n=6) and C – European aspen sites in age 65-131 (n= 6).

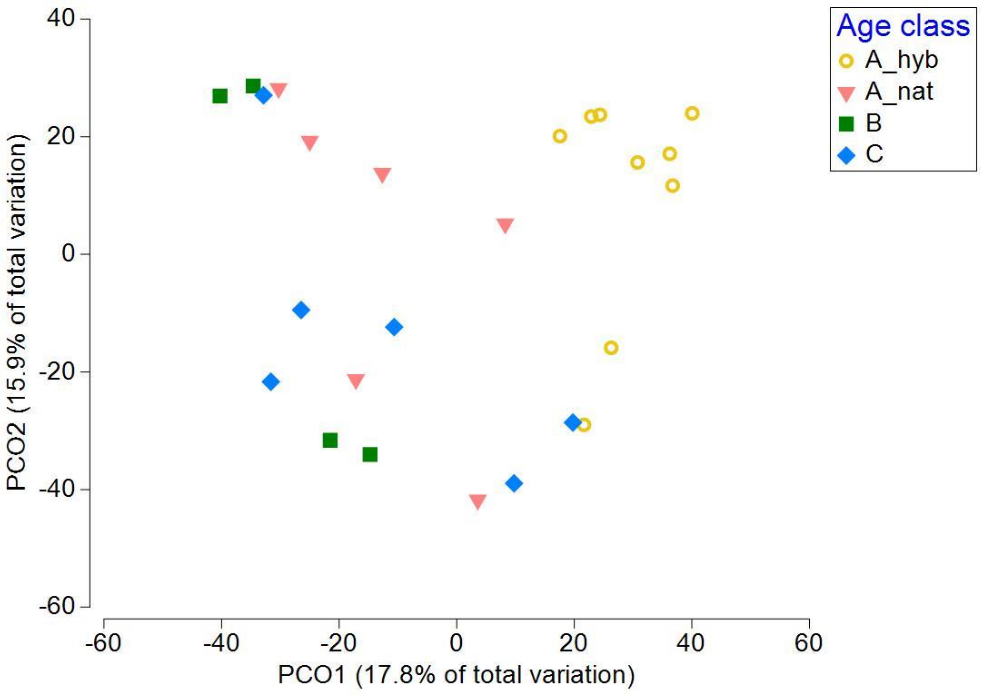

Figure S11. Principal coordinate analysis (PCO) based on the relative abundance of fungal OTUs, describing the variation in AM community structure, classified by the age classes in selected plantation plots (A_hyb=17-18 year old, n=9) and forest (A_nat= forest sites in age 8-29 (n=9); B= forest sites in age 30-55 (n=4) and C – European aspen sites in age 65-131 (n= 6).
